# Variability of multidimensional diffusion–relaxation MRI estimates in the human brain

**DOI:** 10.1162/imag_a_00387

**Published:** 2024-12-11

**Authors:** Eppu Manninen, Shunxing Bao, Bennett A. Landman, Yihong Yang, Daniel Topgaard, Dan Benjamini

**Affiliations:** Multiscale Imaging and Integrative Biophysics Unit, National Institute on Aging, NIH, Baltimore, MD, United States; Department of Electrical and Computer Engineering, Vanderbilt University, Nashville, TN, United States; Neuroimaging Research Branch, National Institute on Drug Abuse, Baltimore, MD, United States; Department of Chemistry, Lund University, Lund, Sweden

**Keywords:** diffusion, relaxation, multidimensional MRI, microstructure, reproducibility, human brain

## Abstract

Diffusion–relaxation correlation multidimensional MRI (MD-MRI) replaces voxel-averaged diffusion tensor quantities and$R_{1}$and$R_{2}$relaxation rates with their multidimensional distributions, enabling the selective extraction and mapping of specific diffusion–relaxation spectral ranges that correspond to different cellular features. This approach has the potential of achieving high sensitivity and specificity in detecting subtle changes that would otherwise be averaged out. Here, the whole brain characterization of MD-MRI distributions and derived parameters is presented and the intrascanner test–retest reliability, repeatability, and reproducibility are evaluated to promote the further development of these quantities as neuroimaging biomarkers. We compared white matter tracts and cortical and subcortical gray matter regions, revealing notable variations in their diffusion–relaxation profiles, indicative of unique microscopic morphological characteristics. We found that the reliability and repeatability of MD-MRI-derived diffusion and relaxation mean parameters were comparable with values expected in conventional diffusion tensor imaging and relaxometry studies. Importantly, the estimated signal fractions of intravoxel spectral components in the MD-MRI distribution, corresponding to white matter, gray matter, and cerebrospinal fluid, were found to be reproducible. This underscores the viability of employing a spectral analysis approach to MD-MRI data. Our results show that a clinically feasible MD-MRI protocol can reliably deliver information of the rich structural and chemical variety that exists within each imaging voxel, creating potential for new MRI biomarkers with enhanced sensitivity and specificity.

## 1 Introduction

Diffusion–relaxation multidimensional MRI (MD-MRI) acquisition integrates meso- and microstructural probes (Callaghan, 2011) with chemical composition sensitivity (Tofts, 2003). While relaxation values can be transformed into estimates of, for example, myelin fraction (Mackay et al., 1994;Vasilescu et al., 1978) and diffusion tensors into estimates of, for example, general orientation of nerve fiber bundles (Basser et al., 1994) within individual millimeter-scale image voxels, achieving a more precise interpretation is hindered by the complex reality that each voxel comprises numerous cells with varying sizes, shapes, and orientations (Livet et al., 2007). This heterogeneity creates considerable uncertainty when establishing connections between the observable metrics and the specific microscopic details we are often interested to discern.

While the classical diffusion tensor imaging (DTI) model (Basser et al., 1994) has proven sensitive to probing brain microstructure in a range of scenarios and applications (Soares et al., 2013), it is encumbered by inherent limitations that impede its applicability and the precision of its interpretations (Novikov et al., 2018). To enhance specificity, various multicomponent biophysical models have been developed and successfully applied to study brain white matter (WM) (Assaf & Basser, 2005;Benjamini et al., 2016;Stanisz et al., 1997;Veraart et al., 2020;Zhang et al., 2012). While these models offer advantages in specificity and interpretability, they rely on strict assumptions and incorporate fixed parameters to reduce fitting ambiguities (Novikov et al., 2018), which may not be valid in disease states or even in heterogeneous gray matter (GM) regions (Jespersen et al., 2019;Lampinen et al., 2019;Novikov et al., 2018;Veraart et al., 2019).

An alternative approach is to avoid a biophysical model and apply a data-driven estimation of distributions of quantitative metrics. This strategy applies a mathematical representation that balances physical accuracy, simplicity, and usefulness in addressing scientific questions without encouraging overinterpretation. First introduced in 1986 to obtain$T_{2}$distributions from muscle tissue (Kroeker & Mark Henkelman, 1986), this approach allows to intuitively expand the dimensionality of the data by simultaneously encoding both diffusion and relaxation. Over the years, it has been used to resolve individual diffusion and relaxation properties of distinct water populations, and their correlations (Hürlimann & Venkataramanan, 2002;Silva et al., 2002). The obtained information has been shown invaluable to the study of tissue microstructure and brain connectivity (de Almeida Martins et al., 2020,^2021^;Kundu et al., 2023;Peled et al., 1999;Reymbaut et al., 2021;Stanisz & Henkelman, 1998;Yon et al., 2020) as well as pathology (Benjamini et al., 2020,^2022^;Kim et al., 2017;Slator et al., 2021;Wei et al., 2022).

An important aspect of translational motion of particles in biological tissue is its time dependency (Callaghan, 2011;Price, 2009). This ensemble translational motion of particles can be analyzed in two ways: in the time domain using metrics like mean-square displacements (Einstein, 1905), apparent diffusivities (Woessner, 1963), and velocity autocorrelation functions (Uhlenbeck & Ornstein, 1930), or in the frequency domain using tensor-valued diffusion spectraD(ω), derived via Fourier transformation (Callaghan & Stepišnik, 1995;Stepišnik, 1981). Deciding between time- and frequency-domain analysis for a specific application is challenging, but recent studies suggest a preference for the frequency-domain approach in*in vivo*human research (Arbabi et al., 2020;Hamilton et al., 2024;Hennel et al., 2021;Maekawa et al., 2020;Tan et al., 2020;Tétreault et al., 2020;Xu et al., 2020,^2021^).

The ω-dependence ofD(ω)is often approximated as Lorentzian, reflecting an exponential velocity autocorrelation function (Stepišnik, 1998;Uhlenbeck & Ornstein, 1930), but more precise expressions exist for simple pore shapes such as parallel planes, cylinders, spheres (Stepišnik, 1993), and for random permeable barriers (Novikov et al., 2014). The latter model, which captures structural disorder without assuming specific geometry, has been increasingly used to analyze*in vivo*human brain data in recent studies (Arbabi et al., 2020;Fieremans et al., 2016;Lee et al., 2020). Further advancing the field, the employment of diffusion acquisition strategies that incorporate tensor-valued diffusion encoding with free gradient waveforms (Westin et al., 2014) contains information about higher order diffusion propagator covariances not present in linear diffusion encoding. Theω-dependence ofD(ω)components can be accessed by using modulated gradientsg(t)(Stepišnik, 1981), which produce tensor-valued encoding spectrab(ω)that concentrate spectral power on specific tensor elements and ω values (Lasič et al., 2022;Lundell & Lasič, 2020). The diffusion tensor distribution (DTD) provides a framework for separating and decoupling distinct water pools within complex and heterogeneous systems (Basser & Pajevic, 2003). Parametric DTD models resolve the mathematical instability in the estimation process; however, they rely on preassumed models for the underlining distribution (Jian et al., 2007;Magdoom et al., 2021). An alternative approach is the nonparametric DTD, which can be reliably estimated from tensor-valued diffusion encoding data, and allows for resolving WM, GM, and CSF from isotropic-anisotropic correlations (de Almeida Martins & Topgaard, 2016;Topgaard, 2019). Recently, a nonparametric tensor-valued Lorentzian diffusion spectra framework has been proposed as a flexible representation to capture MRI signal responses from diverse model systems and biological tissues (Narvaez et al., 2024), distinguishing between restricted and anisotropic diffusion effects. It was shown to reproduce the synthetic data generated by the random permeable barrier model (Novikov et al., 2014).

Demonstrations of nonparametric diffusion–relaxation correlations of MD-MRI includedD-$R_{1}$-$R_{2}$in vivo in humans (Martin et al., 2021), andD(ω)-$R_{1}$-$R_{2}$ex vivo in rat (Narvaez et al., 2022). The latter was recently translated to an efficient and sparse in vivo frequency-dependent MD-MRI acquisition protocol that provides whole brain coverage at 2-mm isotropic resolution (Johnson et al., 2024). This proof-of-concept study tested the feasibility of estimating voxel-wise distributions of frequency-dependent diffusion tensors,D(ω), and longitudinal and transverse relaxation rates,$R_{1}$and$R_{2}$, from this sparsely acquired MD-MRI data. The rich information contained within the high-dimensionalD(ω)-$R_{1}$-$R_{2}$distributions can be distilled into first (means) and second (variances and covariances) moment statistics of, for example, frequency-dependent isotropic diffusivity and diffusion anisotropy,$R_{1}$, and$R_{2}$, in each voxel. Further, having voxel-wise distributions provides access to distinct water populations within a voxel, and, therefore, enables improved specificity toward WM, GM, and cerebrospinal fluid (CSF) (de Almeida Martins et al., 2020). In turn, this unique intravoxel information allows to map microstructure-specific diffusion and relaxation properties without relying on biophysical assumptions or models.

In recent studies, the specificity of estimated intravoxel components using MD-MRI was evaluated in both physical (Benjamini & Basser, 2016;de Almeida Martins & Topgaard, 2016,^2018^) and synthetic (Reymbaut et al., 2020) phantoms, and via comparisons with microscopy of fixed tissue samples from the rat and ferret brain (Benjamini & Basser, 2017;Narvaez et al., 2022). The applicability of this framework for*in vivo*human MRI was confirmed by comparing MD-MRI-derived values in the human brain with those reported in the literature (de Almeida Martins et al., 2021;Martin et al., 2021;Naranjo et al., 2021). In this context, our aims are to present a characterization ofD(ω)-$R_{1}$-$R_{2}$estimates throughout the human brain and to evaluate their intrascanner test–retest reliability and repeatability. These efforts contribute to the ongoing development of the MD-MRI framework as a neuroimaging modality.

## 2 Methods

### 2.1 Participants

Ten healthy participants (mean age 48 years, standard deviation 14.4 years; 4 women) were each scanned twice, a few weeks apart (i.e., a total of 20 scans). Participants were systematically drawn from ongoing healthy cohorts of the National Institute on Drug Abuse (NIDA). Experimental procedures were performed in compliance with our local Institutional Review Board, and participants provided written informed consent. Prior to each scan, NIDA clinical and nursing units conducted COVID-19 testing, urine drug tests, a physical examination, and a questionnaire on pre-existing conditions and daily habits. Exclusion criteria included major medical illness or current medication use, a history of neurological or psychiatric disorders or substance abuse.

### 2.2 Data acquisition

The MRI data acquisition sequences and procedures used were the same as in a previous study (Johnson et al., 2024). Data were acquired using a 3T scanner (MAGNETOM Prisma, Siemens Healthcare AG, Erlangen, Germany) with a 32-channel head coil. MD-MRI data were acquired with 2-mm isotropic voxel size using a single-shot spin-echo EPI sequence modified for tensor-valued diffusion encoding with free gradient waveforms (Martin et al., 2021;Wetscherek et al., 2015). The acquisition parameters were set as follows: FOV = 228 × 228 × 110 mm^3^, voxel size = 2 × 2 × 2 mm^3^, acquisition bandwidth = 1512 Hz/pixel, in-plane acceleration factor 2 using GRAPPA reconstruction with 24 reference lines, effective echo spacing of 0.8 ms, phase-partial Fourier factor of 0.75, and axial slice orientation.

**Fig. 1.**
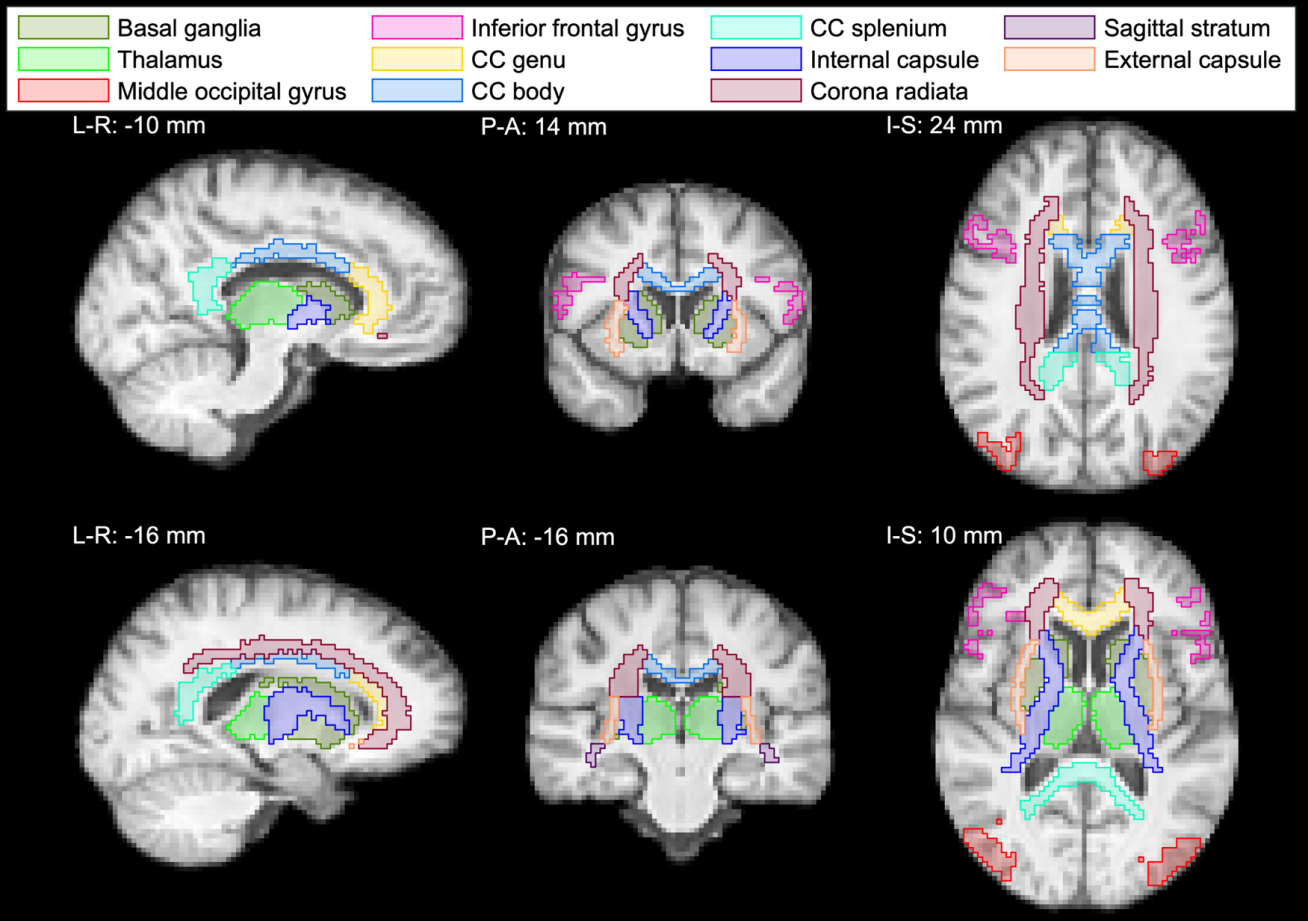
Regions of interest (ROIs) used in the ROI analysis as shown on the cohort-averaged$T_{1}$-weighted template. Subcortical (basal ganglia and thalamus) and cortical (middle occipital gyrus and inferior frontal gyrus) gray matter ROIs were produced using the spatially localized atlas network tiles method (SLANT). Commissural (genu, body, and splenium of the corpus callosum (CC)), projection (internal capsule and corona radiata), and association (sagittal stratum and external capsule) white matter tracts were produced using the Johns Hopkins University diffusion tensor imaging-based white matter atlas. The slice coordinates shown are approximate coordinates in the Montreal Neurological Institute (MNI) space. Abbreviations: L-R, left-right; P-A, posterior-anterior; I-S, inferior-superior.

The acquisition protocol was designed based on previously described heuristic principles (Martin et al., 2021) used to achieve an efficient and sparse MD-MRI acquisition (Johnson et al., 2024). Briefly, in addition to ab=0ms/µm^2^volume, numerically optimized gradient waveforms (Sjölund et al., 2015) that produce linear, planar, and sphericalb-tensors were employed withb-tensor magnitude,b, ranging between 0.1 and 3 ms/µm^2^, andb-tensor normalized anisotropy,$b_{\Delta }$, of – 0.5 (planar), 0 (spherical), and 1 (linear). The spectral content of the employed diffusion gradient waveforms was in the range of 6.6–21 Hz centroid frequencies,$\omega_{\text{cent}}/2\pi$. We note that eachb-tensor contains a range of frequencies and that the centroid frequency is used only as its representative value. All data inversion and metric calculations use frequency as such and do not rely on reducing the spectral content ofb(ω)to a single number. The datasets were acquired with a single phase encoding direction (anterior to posterior, AP), and an additionalb=0ms/µm^2^volume with reversed phase encoding direction (PA). Sensitivity to$R_{1}$and$R_{2}$was achieved by acquiring data with different combinations of repetition times, TR = (0.62, 1.75, 3.5, 5, 7, 7.6) s and echo times, TE = (40, 63, 83, 150) ms. The number of concatenations and preparation scans was increased to allow values of TR below 5 s. A total of 139 individual measurements were recorded over 40 min. More details of the acquisition protocol are available inJohnson et al. (2024), along with a detailed summary of the acquisition protocol shown inSupplementary Figure 1. In addition to MD-MRI, fat-suppressed$T_{1}$-weighted MPRAGE images with TE=3.42 ms, TR=1900 ms, and 1 mm isotropic voxel size were also acquired as structural images. The structural data were utilized in image registrations and region of interest (ROI) segmentations.

### 2.3 MD-MRI data preprocessing

We followed recent recommendations for denoising and preprocessing of sparse MD-MRI data (Johnson et al., 2024). In short, all data volumes were first concatenated and underwent denoising with the MPPCA technique (Veraart et al., 2016). Then, Gibbs ringing correction (Kellner et al., 2016) for partial k-space acquisitions (Lee et al., 2021) was performed, followed by motion and eddy currents distortion corrections using TORTOISE’s DIFFPREP (Rohde et al., 2004) with a physically based parsimonious quadratic transformation model and a normalized mutual information metric. For susceptibility distortion correction, a$T_{1}$-weighted image was initially converted into a$T_{2}$-weighted image withb=0s/mm^2^contrast (Schilling et al., 2019), which was fed into the DR-BUDDI software (Irfanoglu et al., 2015) for AP-PA distortion correction. The final preprocessed images from the MD-MRI sequence were output with a single interpolation in the space of the anatomical$T_{1}$-weighted MPRAGE image at native MD-MRI in-plane voxel size (2 mm isotropic).

### 2.4 Estimation of MD-MRI parameters

Solving the jointD(ω)-$R_{1}$-$R_{2}$probability density distribution from the MD-MRI measurements is an ill-conditioned problem. To solve that inversion problem, the estimation of the MD-MRI parameters used in this study followed the procedures described inNarvaez et al. (2022). The multidimensional diffusion MRI toolbox for MATLAB (Nilsson et al., 2018) was used to perform a Monte Carlo inversion (de Almeida Martins & Topgaard, 2018;Prange & Song, 2009) for the preprocessed MD-MRI data. In the inversion, the MD-MRI signalS(b(ω),TE,TR)is represented as a weighted sum of discrete componentscof the joint probability density distributionD(ω)-$R_{1}$-$R_{2}$:

$$\begin{array}{l} S\left(\text{b}(\omega ),\text{TE},\text{TR}\right)=\sum_{c=1}^{N_{c}}f_{c}\exp\left(-\int_{-\infty }^{\infty }\text{b}(\omega ):{\text{D}}_{c}(\omega )\text{d}\omega \right)\\ \;\;\;\;\;\;\;\;\;\;\;\;\;\;\;\;\;\;\;\;\;\;\;\;\;\;\;\left(1-\exp\left(-\text{TR}\cdot R_{1,c}\right)\right)\exp\left(-\text{TE}\cdot R_{2,c}\right), \end{array}$$

where$N_{c}$is the number of fitted components,$f_{c}$is the weight of the estimated componentc,:denotes a generalized dot product, andD(ω)is an axially symmetric diffusion tensor that depends on the angular frequency of the diffusion gradient waveform. The diffusion tensors are approximated as axisymmetric Lorentzians parameterized by the zero-frequency axial and radial diffusivities [$D_{∥}$,$D_{⊥}$], azimuthal and polar angles [θ,ϕ], high frequency isotropic diffusivity,$D_{0}$, axial and radial transition frequencies, [$\Gamma_{∥}$,$\Gamma_{⊥}$], along with longitudinal and transversal relaxation rates [$R_{1}$,$R_{2}$]. In this study, these parameters were sampled in the ranges$0.05<D_{∥,⊥,0}<5$µm^2^/ms,0<θ<π,0<ϕ<2π,$0.2<R_{1}<2$s^-1^,$1<R_{2}<30$ s^-1^, and$0.01<\Gamma_{∥,⊥}<10000$s^-1^. The Monte Carlo inversion used non-negative least squares with quasi-genetic filtering, bootstrapping, and constraints for the MD-MRI parameter estimates (Benjamini, 2020;de Almeida Martins & Topgaard, 2018). As a result, for each imaging voxel, 300 solutions (bootstraps) of theD(ω)-$R_{1}$-$R_{2}$probability density distribution with up to 10 weighted components (for each bootstrap) were estimated. Voxel-wiseD(ω)-$R_{1}$-$R_{2}$distributions in the primary analysis space [$D_{∥}$,$D_{⊥}$,*θ*,ϕ,$D_{0}$,$\Gamma_{∥}$,$\Gamma_{⊥}$,$R_{1}$,$R_{2}$] were evaluated at selected values ofω/​2π(Narvaez et al., 2024) within the narrow 6.6–21 Hz experimental window, giving a set ofω-dependent distributions in the [$D_{∥}(\omega )$,$D_{⊥}(\omega )$,*θ*,ϕ,$R_{1}$,$R_{2}$] space. It should be noted that the highest frequencies reached for b-values of 0.5, 1.5, and 3 ms/µm^2^were 21, 15, and 11 Hz, respectively. We further chose to express the axial and radial diffusivities as isotropic diffusivity,$D_{\text{iso}}$, and squared normalized anisotropy$D_{\Delta }^{2}$(Conturo et al., 1996).

Two-dimensional projections of the full 6DD(ω)-$R_{1}$-$R_{2}$distributions were constructed to facilitate visualization and to allow investigation of certain ROIs. To obtain the distributions in each voxel, the bootstrap solutions first have to be consolidated into a single representative distribution. This was done using the following procedure: (1) groupingD(ω)-$R_{1}$-$R_{2}$components across different bootstraps solutions using*k*-means clustering over [$D_{iso}(\omega )$,$D_{\Delta }^{2}(\omega )$,*θ*,ϕ,$R_{1}$,$R_{2}$] as features (after normalization according to their respective maximal values) with the*L*1 distance metric. (2) Once grouped, each cluster is transformed into a singleD(ω)-$R_{1}$-$R_{2}$component by taking the weighted mean of each parameter (e.g.,$D_{\text{iso}}$), and the median of the weights. This process then allowed to project and map the median weights of the discrete components from the consolidated distributions onto 64 × 64 meshes in the$D_{\text{iso}}$-$D_{\Delta }^{2}$,$D_{\text{iso}}$-$R_{1}$, and$D_{\text{iso}}$-$R_{2}$planes. These voxel-wise 2D projections were then summed over entire ROIs within each scan, normalized, and finally averaged across all 20 scans to produce characteristic distributions.

The estimated MD-MRI parameters assessed for reliability and repeatability in this study included the expectationE[x](mean) values of isotropic diffusivity$D_{\text{iso}}$($\text{E}[D_{\text{iso}}]),$squared diffusion anisotropy$D_{\Delta }^{2}$($\text{E}\;[D_{\Delta }^{2}]$),$R_{1}$relaxation rate ($\text{E}[R_{1}]$), and$R_{2}$relaxation rate ($\text{E}[R_{2}]$). The effects of restricted diffusion were quantified by a finite difference approximation of the rate of change of the diffusivity metrics with frequencyω/​2πwithin the investigated window (6.6 to 21 Hz), expressed as$\Delta_{\omega \;/2\pi }[x]$of$D_{\text{iso}}$and$D_{\Delta }^{2}$statistics ($\Delta_{\omega /2\pi }\text{ E}\left[D_{\text{iso}}\right]$,$\Delta_{\omega /2\pi }\text{ E}\left[D_{\Delta }^{2}\right]$, respectively) (Johnson et al., 2024;Narvaez et al., 2022). The two-dimensional diffusivity – diffusion anisotropy space can be used to delineate subvoxel components corresponding to different microstructural environments (Pierpaoli et al., 1996;Topgaard, 2019). Subvoxel components were expressed as bin signal fractions,$f_{\text{bin}1}$,$f_{\text{bin}2}$, and$f_{\text{bin}3}$($D_{\text{iso}}<2.5$µm^2^/ms and$D_{\Delta }^{2}>0.25$;$D_{\text{iso}}<2.5$µm^2^/ms and$D_{\Delta }^{2}<0.25$; and$D_{\text{iso}}>2.5$µm^2^/ms and$0<D_{\Delta }^{2}<1$, respectively), which roughly represent WM, GM, and CSF signal fractions, respectively. Intravoxel information from diffusion–relaxation correlations can be directly imaged by resolving the relaxation properties according to the above-defined bins (de Almeida Martins et al., 2020). We, therefore, included in this study the bin-resolved means of the$R_{1}$and$R_{2}$relaxation, for example,$\text{E}{\left[R_{1}\right]}_{\text{bin}1}$,$\text{E}{\left[R_{2}\right]}_{\text{bin}3}$. The expectation valuesE[x]provide estimates of voxel averages of isotropic diffusivity$D_{\text{iso}}$, squared diffusion anisotropy$D_{\Delta }^{2}$, and relaxation rates$R_{1}$and$R_{2}$, and are the closest analogues of mean diffusivity and fractional anisotropy in diffusion tensor imaging and monoexponential$R_{1}$and$R_{2}$relaxation rates in relaxation mapping. The frequency dependence parameters ($\Delta_{\omega /2\pi }[x]$) describe the diffusion spectrum, its rate of change, and how the diffusion parameters depend on the frequency of the diffusion encoding gradients. Specifically,$\Delta_{\omega /2\pi }\text{E}[D_{\text{iso}}]$is more commonly known as$\Delta_{f}\text{ADC}$(Aggarwal et al., 2012) or the diffusion dispersion rate (Xu et al., 2016). The bin fractions$f_{\text{bin}1}$,$f_{\text{bin}2}$,$f_{\text{bin}3}$are computed from the discrete$D_{\text{iso}}$and$D_{\Delta }^{2}$components and, thus, depend on their estimation accuracy. All of the above parameters are computed by taking the median over bootstrap replicas of the estimates.

### 2.5 Assessment of reliability and repeatability

#### 2.5.1 Intraclass correlation coefficient (ICC)

We used the intraclass correlation coefficient (ICC) as a measure of reliability. We used a single-measurement, absolute-agreement, two-way mixed-effects model (ICC(A, 1) in the convention of McGraw and Wong) for the computation (McGraw & Wong, 1996). MATLAB was used for the computation (MATLAB Release R2023a, The MathWorks, Inc., Natick, MA, USA). ICC estimates the proportion of variance due to differences between the participants compared with the total variance of the data. In other words, it is an estimate of the fraction of the total variance that is not due to measurement errors (or errors introduced by the processing pipeline, such as image registration inaccuracy). It characterizes whether differences between the participants can be detected from the noisy measurements. ICC can be improved by either decreasing the measurement error or by increasing the biological variability in the sample, such as by selecting participants with larger differences in age. The ICC ranges from 0 to 1, and while negative values are possible, they are treated as zeros. We use the classification used byKoo and Li (2016)to interpret the ICC values: poor reliability (ICC <0.5), moderate reliability (0.5 ≤ICC <0.75), good reliability (0.75 ≤ICC <0.90), and excellent reliability (ICC ≥0.9).

#### 2.5.2 Within-subject coefficient of variation (CV _ws_ )

We used the within-subject coefficient of variation (CV_ws_) as a measure of repeatability (Luque Laguna et al., 2020). It is the ratio of within-subject standard deviation$\sigma_{ws}$to the mean over subjectsμ. We estimate CV_ws_as

$${\text{CV}}_{ws}\;=\;\;\frac{\sigma_{ws}}{\left|\mu \right|}\approx \frac{\sqrt{{\text{MS}}_{ws}}}{\left|\bar{\mu }\right|},$$

where MS_ws_is the within-subject mean squares and$\bar{\mu }$is the mean over participants and repetitions. Note that we take the absolute value of the mean because some of the MD-MRI parameters we use can take negative values. A within-subject coefficient of variation${\text{CV}}_{ws}=0$indicates perfect repeatability.

### 2.6 Voxel-wise analysis

Discounting super-resolution and biophysical modeling-based approaches, voxel-wise analysis enables the most spatially specific analyses of MR images. To facilitate such an analysis, we registered (transformed) the MR images of the different subjects and time points to a common space (a study-specific template). Because the images are aligned, differences in MR signals between subjects (or time points) can be evaluated at each imaging voxel. However, this approach of analysis is sensitive to image registration inaccuracies and imaging noise. Accuracy of the image registration depends on the resolution, contrast, and signal-to-noise ratio of the images, as well as on the degree of anatomical differences between the subjects. We applied spatial filtering after image registration to alleviate the confounds of image registration inaccuracies and measurement noise on the voxel-wise analyses. Although commonly used in voxel-wise test–retest neuroimaging studies (Caceres et al., 2009;Shou et al., 2013;Somandepalli et al., 2015), spatial filtering of images significantly reduces the effective resolution of the resulting data.

#### 2.6.1 Image registration

To enable the voxel-wise analysis, the MD-MRI parameter maps of the different participants were aligned to a common space using a study-specific template. Advanced Normalization Tools (Avants et al., 2011) (ANTs version 2.4.4,http://stnava.github.io/ANTs/, accessed on 14 June 2023) was used to compute and apply the image registrations. High-resolution (1-mm isotropic)$T_{1}$-weighted MPRAGE images were used to create the study-specific template. First, repetition 1 and repetition 2 of each participant were registered to a mid-way space between the repetitions with an affine transform and averaged to avoid causing interpolation bias that would differ between each repetition (Reuter et al., 2012). Then, a study-specific template was formed from the test–retest average$T_{1}$-weighted images of the different participants using a combination of affine and symmetric image normalization (SyN) transforms. The$T_{1}$-weighted template image (1-mm isotropic resolution) was downsampled to match the resolution of the MD-MRI images (2-mm isotropic resolution). Finally, because the MD-MRI images of each participant had already been aligned to their corresponding$T_{1}$-weighted image during the preprocessing stage (Section 2.3), the MD-MRI parameter maps of each participant and repetition were transformed to the 2-mm resolution template space by combining and applying the image transforms that were computed to make the$T_{1}$-weighted template. Only one interpolation step was applied (after the application of all the image transforms in succession) to avoid the accumulation of image interpolation errors.

#### 2.6.2 Spatial filtering

When image misregistration occurs, a brain structure will be misaligned between different participants. Spatial filtering can be applied to blend together the surroundings of each voxel, thus increasing the degree of overlap in the brain structure between the participants at the cost of effective image resolution. As a side effect, spatial filtering also increases the signal-to-noise ratio of each voxel. After the transformation of the parameter maps to the template space, spatial filtering was applied to reduce the influence of inaccuracies caused by image registration. The choice of the optimal filtering function is nontrivial. However, asCaceres et al. (2009)demonstrated, ICC can be used to guide the decision because it quantifies how well differences between the subjects can be detected over the overall level of variability. Moderate amounts of smoothing can decrease the overall variability (image misalignment and imaging noise), thus increasing the ICC. However, smoothing decreases the spatial specificity of the parametric maps. If there is a regionally specific difference between subjects, it may vanish when excessive smoothing is applied. We applied Gaussian spatial filtering at different values of full width at half maximum (FWHM) for the kernel. When applying the filter, signal contributions from voxels outside of the brain mask were excluded. Then, we computed ICC for each voxel and the median of the ICC values of all brain voxels.Supplementary Figure 2demonstrates varying behaviors of median ICC as a function of the amount of smoothing applied, depending on the MD-MRI parameter map. Some, such as$\text{E}\;[D_{\text{iso}}]$, increase monotonically with the amount of smoothing applied. Such behavior indicates global differences in the parameter values between the participants. Other parameters, such as$\text{E}\left[D_{\Delta }^{2}\right]$, display an optimum at a moderate amount of smoothing, indicating spatially localized differences between the participants that we should seek to preserve. A value of FWHM = 2 pixels (4 mm) was chosen for the voxel-wise analyses, as that value appears to retain such localized differences in all of the parameter maps, yet offers a significant boost to ICC values by decreasing the overall variability due to image misalignment and noise. Values of smoothing FWHM larger than 2 pixels decreased the median voxel-wise ICC values for some parameter maps, suggesting that the decrease in spatial specificity was outweighing the benefits associated with spatial smoothing.

#### 2.6.3 Brain-level statistics of voxel-wise metrics

After the application of spatial filtering (FWHM = 2 pixels = 4 mm), we computed reliability (ICC) and repeatability (CV_ws_) measures for each brain voxel. Violin plots were used to visualize the distributions of voxel-wise values of ICC and CV_ws_over the whole brain. For some MD-MRI parameter maps, a small number of voxels had very high values of CV_ws_. The resulting distributions of voxel-wise values were very broad yet very sparse, making it difficult to produce plots that represented the correct shapes of the distributions. For those distributions of CV_ws_that contained values higher than 100%, the MATLAB function “histcounts” (with automatically selected bins) was used to iteratively remove the voxels that were contained within histogram bins with a density of less than 0.1% of the total number of voxels. The resulting violin plots were labeled to indicate that they contained outliers that were removed prior to plotting. This outlier removal process was applied only for the violin plots of CV_ws_.

### 2.7 ROI-based analysis

In contrast to voxel-wise analysis, ROI-based analysis computes statistical measures within a chosen anatomical region. In our analysis, we compute the mean of each MD-MRI parameter map for each ROI. The resulting parameter estimates are thus spatially averaged based on anatomically delineated brain regions. Compared with voxel-wise analysis, ROI-based analysis is more robust as it is less susceptible to imaging noise and image registration inaccuracies. Voxel-wise analysis has gained popularity as an exploratory method, whereas ROI-based analysis is more common for (anatomically or functionally) hypothesis-driven studies.

#### 2.7.1 Whole brain segmentation

The spatially localized atlas network tiles (SLANT) method was used to perform whole brain segmentation (Huo et al., 2019) and obtain cortical and subcortical GM ROIs. Briefly, SLANT employs multiple independent 3D convolutional networks to segment the brain. Each of the networks is only responsible for a particular spatial region, thus the task of each network is simplified to focus on patches from a similar portion of the brain. Within this end-to-end pipeline, affine registration, N4 bias field correction, and intensity normalization are employed to roughly normalize each brain to the same space before segmentation. After each network performs its duty, the segmentation labels are fused together to form labels for 132 anatomical regions, primarily from subcortical and cortical areas, based on the BrainCOLOR protocol (https://mindboggle.info/braincolor/). The SLANT framework has shown high intra- and interscan protocol reproducibility (Xiong et al., 2019). It was performed using the 1-mm-resolution$T_{1}$-weighted MPRAGE images to obtain GM segmentations in the native space of each participant and repetition. Finally, GM segmentations were downsampled to 2-mm resolution to match the resolution of the MD-MRI scans.

The Johns Hopkins University DTI-based WM atlas (https://identifiers.org/neurovault.collection:264) was used to generate segmentations of white matter tracts. First, by using the participant-to-cohort average image transforms computed earlier (seeSection 2.6.1), we formed a cohort-averaged$\text{E}\;[D_{\Delta }^{2}]$image (2-mm isotropic resolution). Then, we registered that image to the 2-mm resolution fractional anisotropy image of the WM atlas. Finally, we applied the inverses of the computed transforms (participant to cohort average and cohort average to WM atlas) to transform the ROIs of the WM atlas to the native space of each participant and repetition.

The segmented ROIs we focused on in our ROI analysis are shown in study-specific template space inFigure 1. They covered different regions of the brain and included subcortical GM regions (basal ganglia and thalamus), cortical GM regions (middle occipital gyrus and inferior frontal gyrus), commissural tracts (genu, body, and splenium of the corpus callosum (CC)), projection tracts (internal capsule and corona radiata), and association tracts (sagittal stratum and external capsule). For each ROI that had a separate ROI for the left and right hemisphere of the brain, we combined the left and the right side to create a single ROI. To ascertain that each image voxel only belonged to one ROI, we removed from the GM ROIs those voxels that belonged to any of the WM ROIs. Finally, we removed all voxels with$f_{\text{bin}3}>0.2$(estimated CSF signal fraction > 0.2) from the ROIs. Some of the ROIs, shown for the different repetitions and participants in native space, are shown inSupplementary Figure 3.

#### 2.7.2 Estimation of ICC and CV _ws_

The estimation of ICC (reliability) and CV_ws_(repeatability) values for each ROI and MD-MRI parameter map was performed in the native space of each participant and repetition to avoid unnecessary image interpolation. We employed an approach typical to ROI analysis, that is, we averaged the parameter value over the voxels within each ROI. Then, ICC and CV_ws_were computed from those ROI-averaged parameter values. Thus, we get estimates for the reliability and repeatability of ROI-averaged MD-MRI parameter maps. Spider (radar) plots were used to visualize differences in MD-MRI parameter values, ICC, and CV_ws_between different ROIs (modified MATLAB function spider_plot,https://github.com/NewGuy012/spider_plot, accessed Aug 28, 2024).

#### 2.7.3 Bland–Altman plots

Bland–Altman plots (Bland & Altman, 1986) were used to assess the presence of a bias between the test and retest measurement for each MD-MRI parameter map. In a Bland–Altman plot, the difference between the test and retest measurement is plotted as a function of the mean of the test and the retest measurement for each data point (in our case, each voxel). To achieve this, the parameter maps and ROIs of each participant and repetition were transformed to the cohort template space. In the cohort template space, a template ROI was formed by the majority vote of the ROIs of the participants and repetitions (at least 50% agreement between the participants and repetitions was needed for a voxel to belong to a certain ROI). Each voxel in the cohort template space that belonged to any of the ROIs included in our ROI analysis was included as a data point in the Bland–Altman plot, for each participant, resulting in 152620 data points (15262 voxels, 10 participants). The MATLAB class “densityScatterChart” (https://github.com/MATLAB-Graphics-and-App-Building/densityScatterChart, accessed July 17, 2023) was used to plot the density scatter plots. The mean of the test–retest difference over all data points (voxels and participants), along with the 95% confidence interval of the observations, was also computed and plotted for each MD-MRI parameter. Finally, we reported the relative mean test–retest difference as a percentage of the grand mean (mean over repetitions, participants, and voxels).

## 3 Results

### 3.1 Profiling of white matter, cortical, and subcortical regions in the human brain

Leveraging the rich information content of the voxel-wiseD(ω)-$R_{1}$-$R_{2}$distributions, we first provide robust microstructural profiling across various brain regions, based on the average data from the 20 scans in our study. The ROIs used in this study are shown inFigure 1, and projections from fullD(ω)-$R_{1}$-$R_{2}$distributions in six representative ROIs, comprising two cortical and two subcortical regions, along with two WM tracts are shown inFigure 2.

**Fig. 2.**
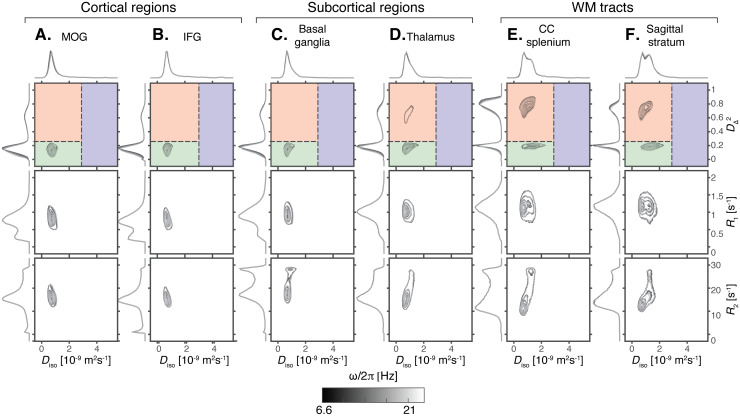
Projections of estimatedD(ω)-$R_{1}$-$R_{2}$distributions in different regions of interest (ROI). The displayed 2D projections include$D_{\text{iso}}$-$D_{\Delta }^{2}$(top row),$D_{\text{iso}}$-$R_{1}$(middle row), and$D_{\text{iso}}$-$R_{2}$(bottom row) distributions. Different grayscale contour lines represent different centroid frequencies for the diffusion encoding gradients (ω/​2π=6.6−21Hz). Colored areas in the$D_{\text{iso}}$-$D_{\Delta }^{2}$distribution plots represent bins that approximately correspond to white matter (red bin), gray matter (green bin), and cerebrospinal fluid (blue bin). Each distribution was first clustered across bootstraps, averaged over all voxels within each ROI, then averaged over the 10 participants and 2 repetitions. (A) Middle occipital gyrus (MOG), (B) inferior occipital gyrus (IFG), (C) basal ganglia, (D) thalamus, (E) splenium of the corpus callosum (CC), (F) sagittal stratum.

For each ROI, the distributions are visualized as projections onto the 2D$D_{\text{iso}}$-$D_{\Delta }^{2}$,$D_{\text{iso}}$-$R_{1}$, and$D_{\text{iso}}$-$R_{2}$planes for 5 frequenciesω/​2πbetween 6.6 and 21 Hz. The well-known differences in tissue microstructure are most clearly manifested in the 2D$D_{\text{iso}}$-$D_{\Delta }^{2}$projection (Topgaard, 2019), which is, therefore, used to define three subvoxel bins, as illustrated by the red, green, and blue overlays inFigure 2. The bin signal fractions,$f_{\text{bin}1}$,$f_{\text{bin}2}$, and$f_{\text{bin}3}$, evaluated at the lowest frequency (ω/​2π= 6.6 Hz), are obtained by integrating over these spectral regions. Additional 2D projections that display the 2D$D_{\Delta }^{2}$-$D_{\text{iso}}$,$D_{\Delta }^{2}$-$R_{1}$, and$D_{\Delta }^{2}$-$R_{2}$(i.e., the anisotropy as a function of relaxation) are shown inSupplementary Figure 4to allow for a more complete investigation of the specific microstructural features of different brain regions.

The distributions shown inFigure 2reflect the microstructural differences of the respective regions and their subvoxel tissue components, revealing differences in composition between cortical and subcortical GM, and WM. Specifically for the 2D$D_{\text{iso}}$-$D_{\Delta }^{2}$projections, cortical GM regions such as the inferior frontal gyrus are mainly composed of morphologically isotropic tissue elements with low diffusivity (Fig. 2A-B, green shading). Subcortical regions such as the thalamus present a wider distribution of tissue elements in terms of both their microscopic shape and size, with an additional highly anisotropic component (Fig. 2D, red shading); and WM tracts such as the splenium of the corpus callosum are mainly composed of morphologically anisotropic and low diffusivity tissue elements (Fig. 2E-F).

The$R_{1}$and$R_{2}$relaxation rates can complement the microstructural picture with compositional information. The 2D$D_{\text{iso}}$-$R_{1}$(Fig. 2) and$D_{\Delta }^{2}$-$R_{1}$(Supplementary Fig. 4) projections show a gradual increase in the$R_{1}$value of the main component from the cortical GM to the subcortical GM to the WM regions. A low$R_{1}$peak (~0.4 s^-1^) was observed in the cortical regions, visible on the 1D$R_{1}$projection. In the 2D$D_{\text{iso}}$-$R_{2}$and$D_{\Delta }^{2}$-$R_{2}$projections, distinct and more heterogeneous trends are observed compared with$R_{1}$, with a single$R_{2}$population in the cortical regions, compared with two$R_{2}$populations in the subcortical and WM regions. From the$D_{\text{iso}}$-$R_{2}$correlations (Fig. 2), both fast- and slow-relaxing$R_{2}$components have qualitatively similar$D_{\text{iso}}$values. On the other hand, from the$D_{\Delta }^{2}$-$R_{2}$correlations (Supplementary Fig. 4), regional differences were observed in which a high anisotropy fast-relaxing population was only observed in the CC splenium, while in the basal ganglia, the thalamus, and the sagittal stratum, only low anisotropy fast-relaxing and high anisotropy slow-relaxing components have been observed.

Diffusion frequency dependency is shown as grayscale contours inFigure 2, and can be more readily observed from the 1D projections in each dimension. Although some frequency dependency can be seen for$D_{\text{iso}}$, it is more pronounced when examining$D_{\Delta }^{2}$. In all regions, frequency dependence of the microscopic anisotropy behaves in a similar manner, in which$D_{\Delta }^{2}$shifts toward lower values with increased frequency.

The voxel-wise multidimensional distributions can be used in different ways to extract information of interest. The most straightforward approach to achieve dimensionality reduction involves computing the per-voxel statistics, such as meansE [x], for the different dimensions, assessed at one of the probed diffusion frequencies. This way, the means$\text{E}[D_{\text{iso}}]$,$\text{E}[R_{1}]$, and$\text{E}[R_{2}]$correspond to conventional mean diffusivity (Basser et al., 1994), quantitative$R_{1}$and$R_{2}$(Weiskopf et al., 2021), respectively. TheE [DΔ2]map is comparable with metrics used to quantify microscopic diffusion anisotropy (Lasič et al., 2014;Lawrenz et al., 2010;Shemesh et al., 2016). Expressing the diffusion frequency dependence, rate of change with frequency (within the investigated range of 6.6–21 Hz) for the per-voxel means and variances can be used (Narvaez et al., 2024). Thus, positive values of the$\Delta_{\omega /2\pi }\text{ E}[D_{\text{iso}}]$parameter indicate diffusion time dependency behavior suggestive of restriction (Aggarwal et al., 2012), while decreased anisotropy with higher frequency results in negative values in the$\Delta_{\omega /2\pi }\text{ E}\;[D_{\Delta }^{2}]$map. These MD-MRI diffusion–relaxation parameters, averaged within the ROIs inFigure 2, are presented inFigure 3. The mean values agree with the expected microstructural differences between GM and WM regions, and also correspond with the observed trends of the 2D projections inFigure 2.

**Fig. 3.**
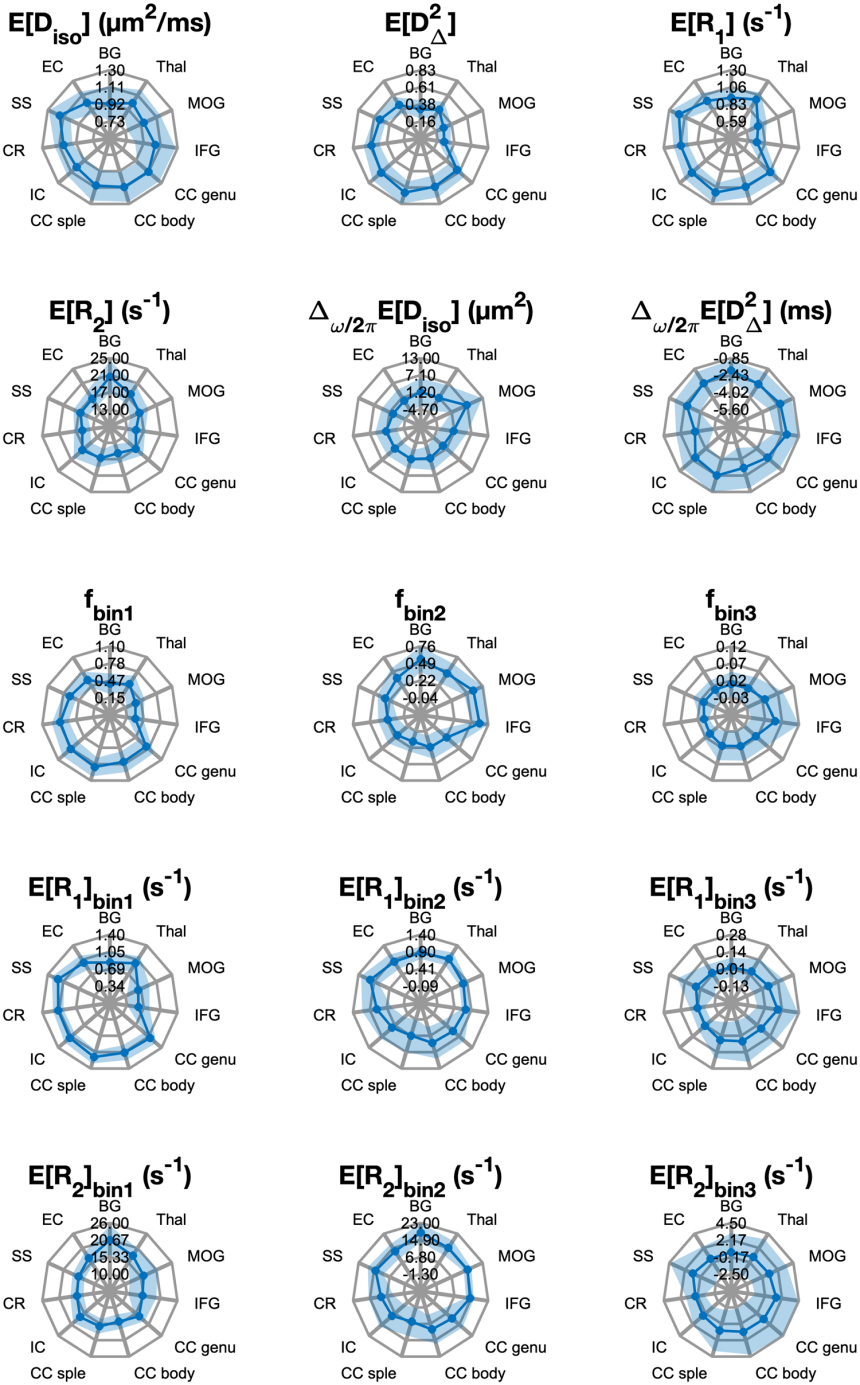
Mean (solid line) and standard deviation (shaded area covers the range of ±1 standard deviation) of region-of-interest-averaged multidimensional MRI parameters. Abbreviations: BG, basal ganglia; Thal, thalamus; MOG, middle occipital gyrus; IFG, inferior frontal gyrus; CC genu, genu of the corpus callosum; CC body, body of the corpus callosum; CC splenium, splenium of the corpus callosum; IC, internal capsule; CR, corona radiata; SS, sagittal stratum; EC, external capsule.

An alternative way to extract information from the high dimensional voxel-wise data is to perform integration across a predefined parameter range, akin to a “spectral” ROI. The obtained integral value, ranging from 0 to 1, indicates the signal fraction within a particular multidimensional distribution. This voxel-wise computation enables the creation of an image reflecting the selected spectral component (Benjamini & Basser, 2020;Benjamini, Bouhrara, et al., 2021;de Almeida Martins et al., 2021;Martin et al., 2021). In this study, we used the previously suggested spectral ROIs shown inFigure 2(de Almeida Martins et al., 2020;Topgaard, 2019), to summarize the WM, GM, and CSF contributions. Voxel-wise signal fractions, averaged within the ROIs inFigure 2, are also presented inFigure 3. We can further leverage the derived subvoxel information from the$D_{\text{iso}}$-$D_{\Delta }^{2}$plane bins illustrated inFigure 2(i.e.,$f_{\text{bin}1}$,$f_{\text{bin}2}$, and$f_{\text{bin}3}$) and use it to resolve relaxation parameters based on their diffusion characteristics (de Almeida Martins et al., 2020;Reymbaut et al., 2021). Summarized values expressing diffusion–relaxation correlation via the (diffusion-) bin-resolved means of$R_{1}$and$R_{2}$relaxation properties are captured with the bin-resolved mean relaxation metrics, for example,$\text{E}{\left[R_{1}\right]}_{\text{bin}1}$,$\text{E}{\left[R_{2}\right]}_{\text{bin}2}$, which can also be found inFigure 3.

To complement the data inFigure 3, numerical values for the means and standard deviations from all ROIs of each MD-MRI parameter, computed over all participants, repetitions, are given inSupplementary Figure 5.

### 3.2 Test–retest reliability and repeatability

We hypothesized that*in vivo*human brainD(ω)-$R_{1}$-$R_{2}$MD-MRI parameter estimates acquired using a clinically achievable protocol are repeatable and reliable. Below, we evaluate this hypothesis for two prevalent image analysis approaches: voxel-wise and ROI-based. We employ the intraclass correlation coefficient (ICC) to assess the reliability, the within-subject coefficient of variation (CV_ws_) to quantify the repeatability, and Bland–Altman plots to estimate the bias of the MD-MRI parameter maps.

#### 3.2.1 Voxel-wise analysis

**Fig. 4.**
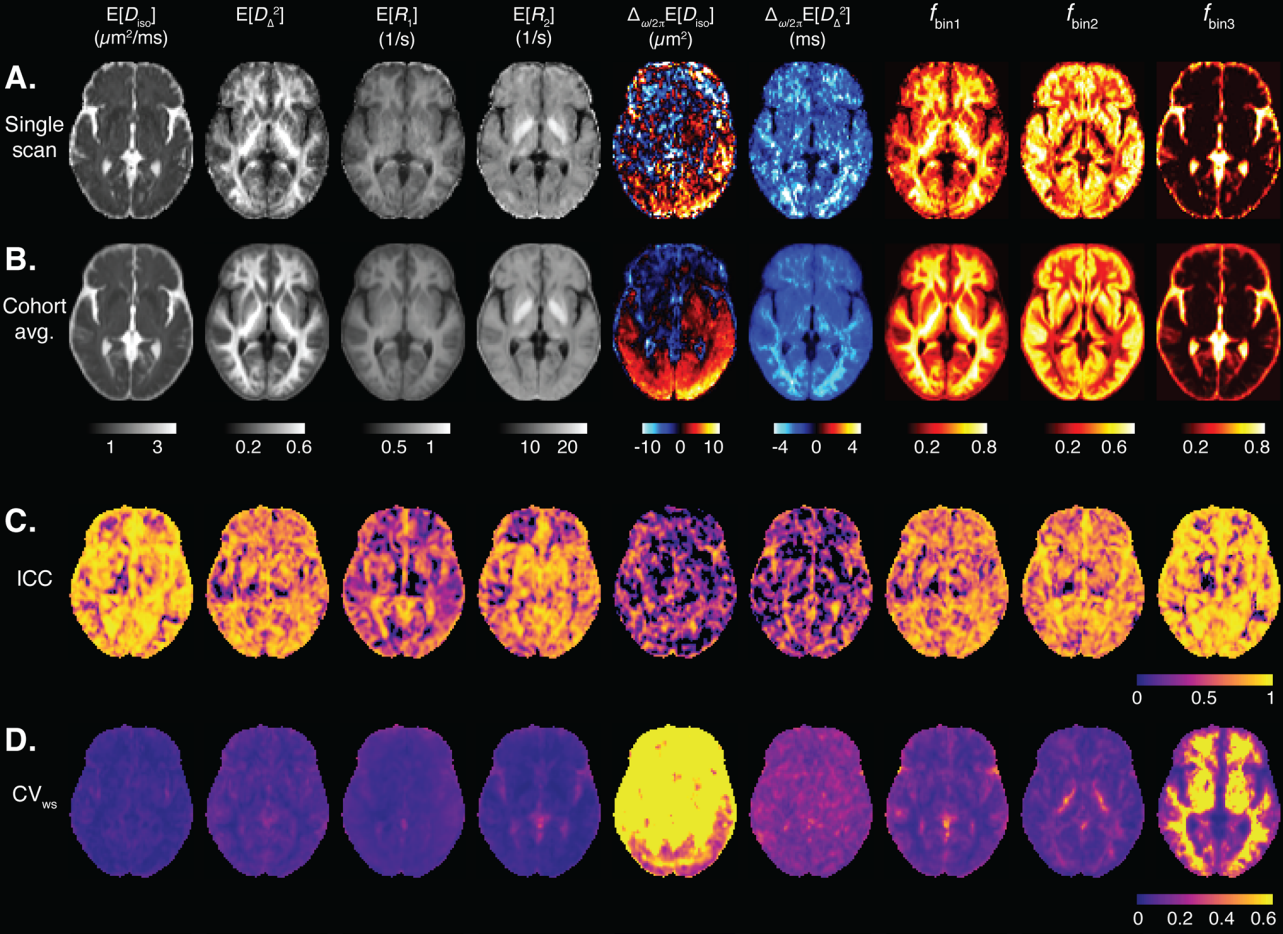
Voxel-wise MD-MRI parameter meansE[x]and signal fraction maps, and their reliability and repeatability measures. MeansE[x]and signal fraction maps, shown for (A) a representative single participant, and for a (B) cohort average. Voxel-wise (C) intraclass correlation (ICC; reliability) and (D) within-subject coefficient of variation (CV_ws_; repeatability), computed for each parameter map. Spatial smoothing was applied to each map before the ICC and CV_ws_computations. The ICC and CV_ws_values demonstrate good reliability and repeatability for the first order statistical parametersE[x]of$D_{\text{iso}}$,$D_{\Delta }^{2}$,$R_{1}$, and$R_{2}$, and low reliability and repeatability for the dependence on diffusion gradient frequency$\Delta_{\omega /2\pi }[x]$of the diffusion parameters. The bin signal fractions$f_{\text{bin}1}$and$f_{\text{bin}2}$demonstrate high reliability and repeatability. The bin signal fraction$f_{\text{bin}3}$shows low repeatability in white matter but high reliability and repeatability elsewhere.

We transformed MD-MRI parameter maps to the cohort template space and estimated ICC (reliability) and CV_ws_(repeatability) values for each brain voxel, after applying spatial filtering to the maps to reduce the effects of image misalignment. Parametric maps of mean values and signal fractions are shown inFigure 4.Figure 4Ashows a representative image slice of the MD-MRI parameter maps from a single subject. The maps are shown after transformation to the cohort template space but without the application of spatial filtering to give a representative example of the image quality.Figure 4Bshows the cohort averages (10 participants, 2 repetitions) for those MD-MRI parameter maps. A representative slice for the MD-MRI parameter maps from all the participants in the study is given inSupplementary Figure 6to visualize the intersubject variability. For each MD-MRI parameter, voxel-wise ICC and CV_ws_values are shown inFigure 4C and 4D, respectively. These were computed after applying a Gaussian spatial filter to the maps (FWHM = 2 pixels = 4 mm).Supplementary Figure 7shows ICC and CV_ws_values computed with no spatial filtering applied to the parameter maps. The subvoxel spectral information can be further utilized to resolve relaxation parameters based on their diffusion characteristics, which are depicted inFigure 5. Each map utilizes two orthogonal scales: the brightness intensity indicates the relative signal fraction, while the color scale denotes the specific relaxation property value. Voxel-wise ICC and CV_ws_values are shown inFigure 5C and 5D, respectively.

**Fig. 5.**
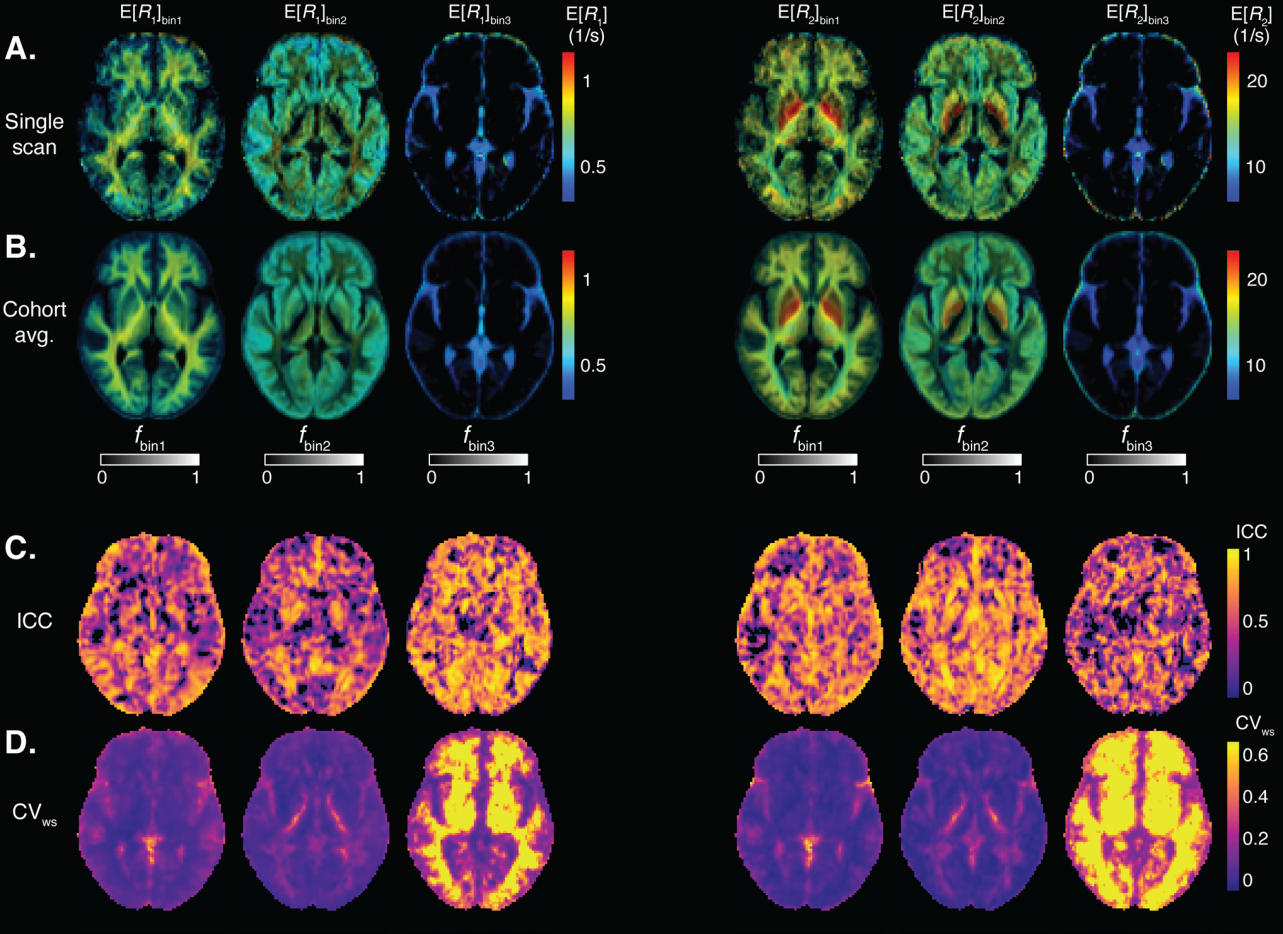
Voxel-wise MD-MRI parameter for bin-resolved relaxation values, and their reliability and repeatability measures. MD-MRI parameter$R_{1}$and$R_{2}$means, resolved according to their respective diffusion bin fractions, shown for (A) a representative single participant, and for (B) a cohort-average. Voxel-wise (C) intraclass correlation coefficient (ICC; reliability) and (D) within-subject coefficient of variation (CV_ws_; repeatability), computed for each parameter map. Spatial smoothing was applied to each map before the ICC and CV_ws_computations. The ICC and CV_ws_values demonstrate good reliability and repeatability except for$f_{\text{bin}3}$, which shows low repeatability in white matter but high reliability and repeatability elsewhere.

**Fig. 6.**
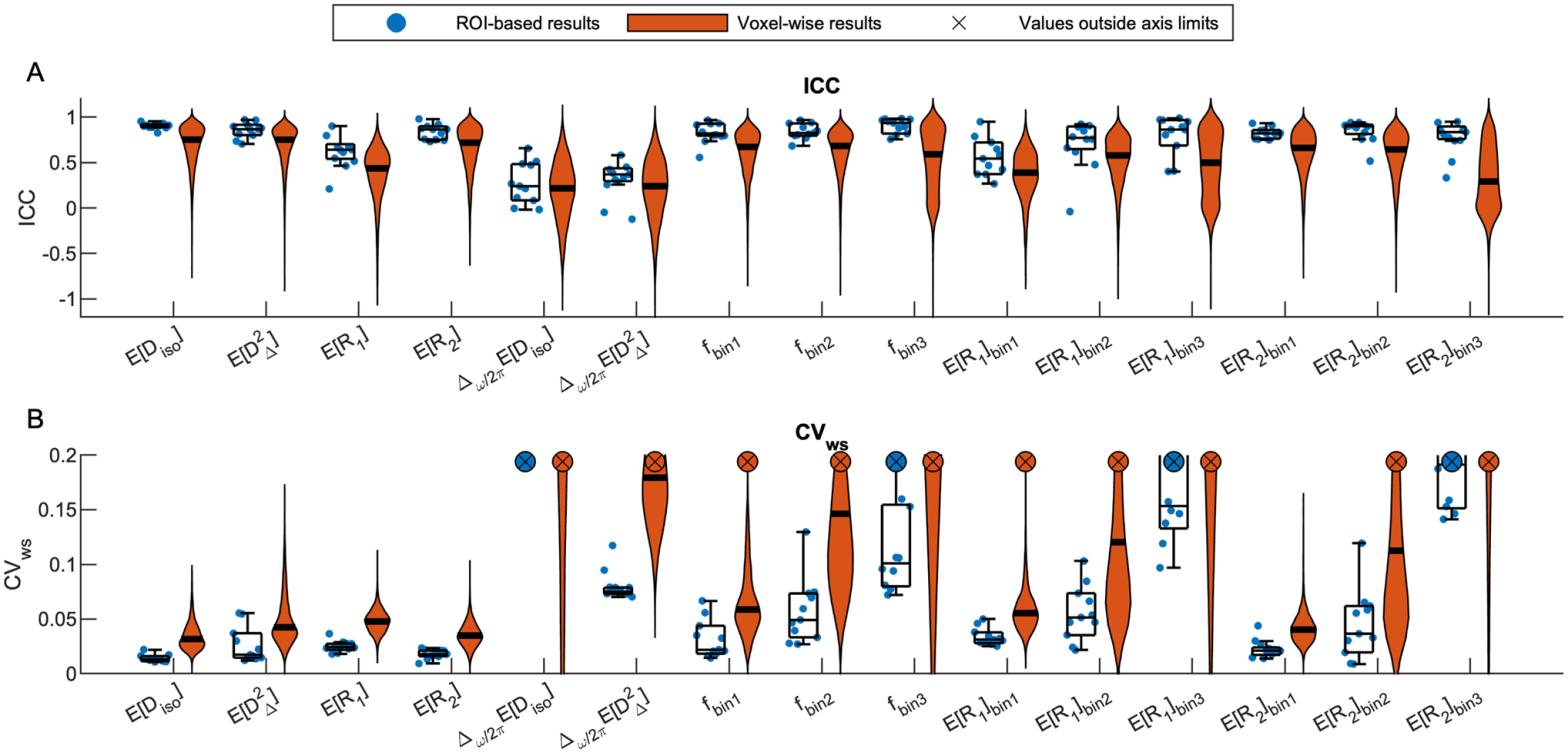
Summary of region of interest (ROI)-based and voxel-wise (A) reliability (intraclass correlation coefficient, ICC) and (B) repeatability (within-subject coefficient of variation, CV_ws_) results. For ROI-based results (blue dots), ICC and CV_ws_were computed for each ROI-averaged multidimensional (MD)-MRI parameter map, and a boxplot was plotted for the collection of ROI-wise results. Voxel-wise ICC and CV_ws_values were plotted as violin plots (orange). Crosses (x) indicate that the parameter in question contained values outside of the shown axis limits (${\text{CV}}_{\text{ws}}>0.20$).

Figure 6shows the distributions of voxel-wise ICC and CV_ws_values for each MD-MRI parameter map. With most MD-MRI parameter maps, high ICC values (high reliability) corresponded to low CV_ws_values (high repeatability), as could be expected.$\text{E}\;[D_{\text{iso}}]$,$\text{E}\;[D_{\Delta }^{2}]$, and$\text{E}[R_{2}]$exhibited both high ICC values and low CV_ws_values.$\text{E}[R_{1}]$showed one of the lowest CV_ws_values but also low ICC values, suggesting low relative measurement error but also small biological variability between the participants. The estimated signal fraction parameter$f_{\text{bin}3}$had relatively high ICC but high CV_ws_values as well. The high CV_ws_values of$f_{\text{bin}3}$were concentrated in WM regions. In those regions,$f_{\text{bin}3}$values are close to 0, and so even minute differences in$f_{\text{bin}3}$between repetitions lead to high CV_ws_values. We also estimated the distributions of the ICC and CV_ws_values over all brain voxels.Supplementary Figure 8shows the distributions of ICC and CV_ws_values in the brain with different levels of spatial filtering applied to the parameter maps.

#### 3.2.2 ROI analysis

The ROIs used in the analysis are shown inFigure 1. Within each ROI and for each participant and repetition, the mean MD-MRI parameter map was computed, from which ICC (reliability) and CV_ws_(repeatability) were estimated. As the parameters are averaged over the ROIs, the number of voxels in each ROI affects the quality of the metrics. All ROIs in this study had comparable volume (~1500 voxels), with the exception of the corona radiata (~3500 voxels) and the sagittal stratum (~500 voxels). The number of voxels for each participant and repetition in each ROI is shown inSupplementary Figure 9.

**Fig. 7.**
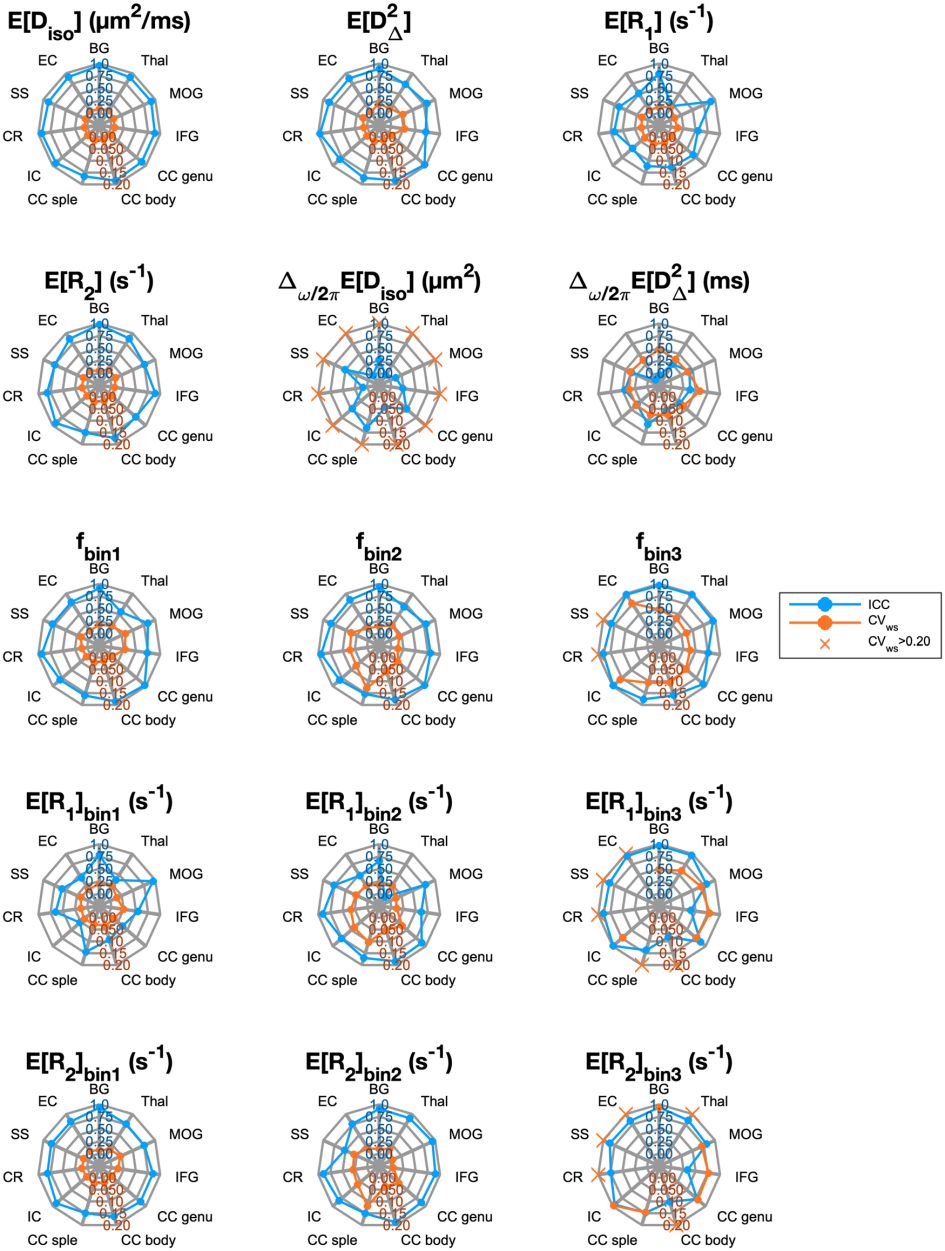
Reliability (intraclass correlation coefficient, ICC; blue line and axis ticks) and repeatability (within-subject coefficient of variation, CV_ws_; red line and axis ticks) of region-of-interest-averaged MD-MRI parameter maps. CV_ws_>0.20values are marked as crosses on the CV_ws_=0.20line. Generally, ROI-based ICC values were higher and CV_ws_values lower than those of individual voxels (Fig. 6). That was expected due to the increase in signal-to-noise ratio achieved through averaging. Abbreviations: BG, basal ganglia; Thal, thalamus; MOG, middle occipital gyrus; IFG, inferior frontal gyrus; CC genu, genu of the corpus callosum; CC body, body of the corpus callosum; CC splenium, splenium of the corpus callosum; IC, internal capsule; CR, corona radiata; SS, sagittal stratum; EC, external capsule.

Reliability and repeatability values for different MD-MRI parameter maps and ROIs are plotted inFigure 7, and their numerical values are given inSupplementary Figure 10.Figure 7allows to visually assess uniformity of the reliability and repeatability across the examined ROIs for individual MD-MRI parameters. Boxplots over all ROIs for the ROI-based ICC and CV_ws_values, shown inFigure 6, indicate, as expected, that ROI-averaged MD-MRI parameters are more reliable and repeatable than their voxel-wise counterparts. The relationship between high reliability (high ICC) and high repeatability (low CV_ws_) in the ROI-based analysis was similar to the results of the voxel-wise analysis, except in the case of$f_{\text{bin}3}$. The reliability (ICC) of$f_{\text{bin}3}$benefited more from the ROI averaging than the other MD-MRI parameters, achieving the highest median ROI-based reliability (ICC = 0.94) out of all the MD-MRI parameters even though its median repeatability was the 4^th^worst (CV_ws_= 0.106). As in the voxel-wise analysis, the ROI-averaged values of$\text{E}[D_{\text{iso}}]$,$\text{E}\;[D_{\Delta }^{2}]$, and$\text{E}[R_{2}]$displayed both high reliability and high repeatability.

**Fig. 8.**
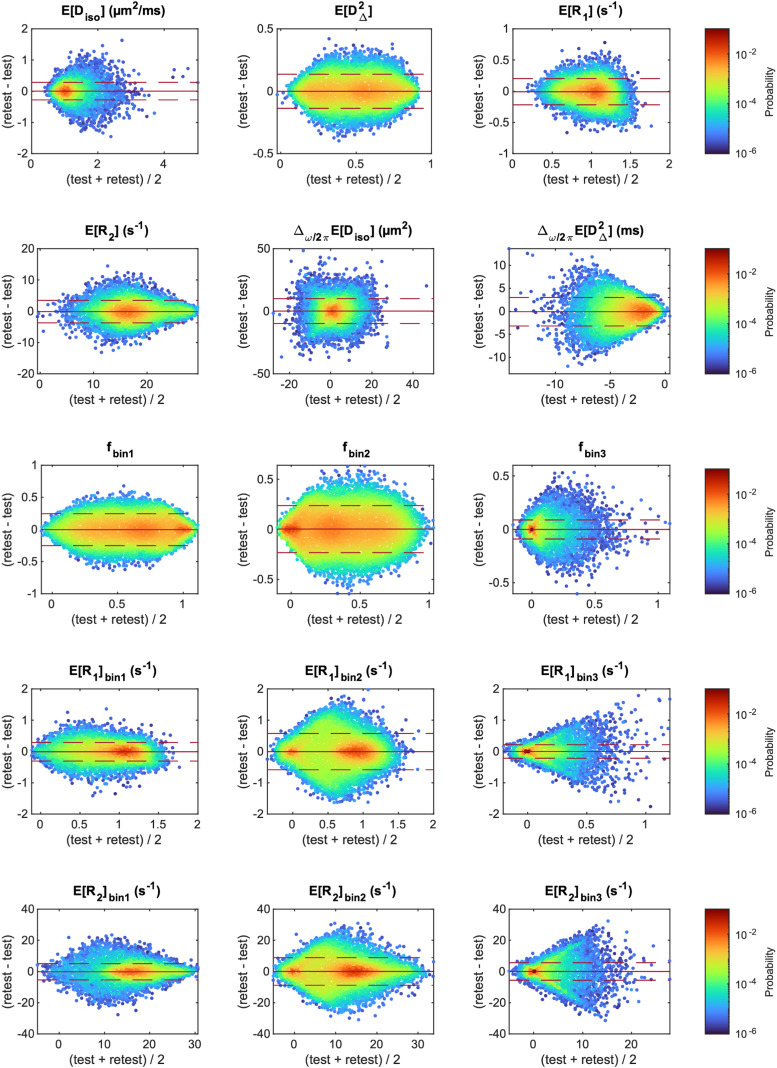
Bland–Altman density plots for the different MD-MRI parameter maps. In each plot, all the voxels that were included in the region of interest analysis are included as data points, for all participants. The mean (solid red line) and the 95% confidence interval of the observations (1.96 ×standard deviation; dashed red line) are shown for each plot.

We assessed the presence of bias between the test and the retest measurement using Bland–Altman plots comprising the different participants and the voxels included in the analyzed ROIs (Fig. 8). For each of the MD-MRI parameters, the 95% confidence interval for the observations included the zero-bias level. The relative mean differences between the test and the retest measurement for the different parameters were$\text{E}\;[D_{\text{iso}}]$: 0.55%,$\text{E}\;\;[D_{\Delta }^{2}]$: -0.031%,$\text{E}[R_{1}]$: -0.66%,$\text{E}[R_{2}]:$-0.65%,$\Delta_{\omega /2\pi }\text{E}\;[D_{\text{iso}}]$: 11%,$\Delta_{\omega /2\pi }\text{E}\;[D_{\Delta }^{2}]$: 3.4%,$f_{\text{bin}1}$: -0.60%,$f_{\text{bin}2}$: 1.1%,$f_{\text{bin}3}$: -1.7%,$\text{E}{\left[R_{1}\right]}_{\text{bin}1}$: -0.84%,$\text{E}{\left[R_{1}\right]}_{\text{bin}2}$: -0.51%,$\text{E}{\left[R_{1}\right]}_{\text{bin}3}$: 2.0%,$\text{E}{\left[R_{2}\right]}_{\text{bin}1}$: -0.96%,$\text{E}{\left[R_{2}\right]}_{\text{bin}2}$: 0.51%,$\text{E}{\left[R_{2}\right]}_{\text{bin}3}$: 6.0%. Those biases were, on average, about one order of magnitude smaller (range: 4 to 140 times smaller) than the median voxel-wise within-subject coefficients of variation CV_ws_(Supplementary Fig. 5).

## 4 Discussion

Diffusion–relaxation correlation MD-MRI is a method that allows to acquire rich information about tissue microstructure by exploring the correlations between different MRI contrasts. This framework replaces voxel-averaged quantities with multidimensional distributions of those quantities that allows to selectively extract and map specific diffusion–relaxation spectral ranges. As a result, it has the potential of achieving high sensitivity and specificity toward detecting subtle changes that would have been otherwise averaged out. The purpose of this work was to methodologically characterize different brain regions in terms of their multicomponent diffusion–relaxation properties, interpret differences and similarities between them, and to assess the framework’s overall repeatability. We compared WM tracts, cortical GM, and subcortical GM regions, and found pronounced differences in their diffusion–relaxation profile, reflecting distinct diversities in their microscopic morphological content. We then applied a test–retest paradigm to estimate the repeatability and reliability of parameters derived from the 40-min whole brain*in vivo*MD-MRI framework, and showed them to be comparable with known DTI reproducibility.

Results from comprehensive profiling of representative WM, cortical GM, and subcortical GM regions are shown inFigure 2,Supplementary Figure 4, andFigure 3. Given the extensive history of DTI studies, it is not surprising that the MD-MRI data allowed to easily differentiate between WM and GM regions based on their different diffusion anisotropy distributions. However, we demonstrated here that meaningful differences can be gleaned even between WM tracts: compared with the sagittal stratum, the CC splenium showed a reduced content of low anisotropy – slow diffusivity components (i.e.,$f_{\text{bin}2}$, seeFigure 3andSupplementary Fig. 5), and had, in addition, a higher content of a fast-relaxing$R_{2}$component. Further, this fast-relaxing$R_{2}$population displayed high microscopic anisotropy. The observed differences in the$R_{2}$components and in$\text{E}{\left[R_{2}\right]}_{\text{bin}1}$can be attributed to the higher myelin content in the CC splenium compared with the sagittal stratum (Dvorak et al., 2021), which would appear as a fast-relaxing microscopically anisotropic peak. The differences in the diffusive properties may be driven by the layered structure of the sagittal stratum (Di Carlo et al., 2019) that makes it more microstructurally heterogeneous compared with the CC.

Our findings in GM revealed distinct microstructural attributes that differentiate between subcortical and cortical regions. Compared with the cortical regions, the thalamus and basal ganglia contained a higher content of microscopically anisotropic components (i.e.,$f_{\text{bin}1}$, seeFig. 3andSupplementary Fig. 5), and a wider distribution of diffusivities, skewed toward larger values. These differences in microscopic anisotropy and in diffusivity are likely to be driven, respectively, by the presence of fiber tracts and the relatively large subcortical nuclei (Morel, 2007). In addition to the diffusion properties, cortical and subcortical GM regions showed differences in their$R_{2}$distributions (seeFig. 2), in which the more microstructurally heterogeneous subcortical regions displayed a fast-relaxing$R_{2}$peak that may be modulated by the presence of macromolecules and myelin lipids.

While we were able to apply the MD-MRI framework to perform a microstructural study of the human brain thus demonstrating its potential, there are two factors that may introduce unwanted variability to its outcomes: the acquisition parameter space is sparsely sampled to keep the scan time feasible, and the parameter estimation is an ill-posed inverse problem. Until now, there has been no evaluation of MD-MRI parameter reproducibility in the living human brain. Therefore, we investigated the reliability (ICC) and repeatability (CV_ws_) of estimated MD-MRI parameters using a test–retest paradigm. The CV_ws_quantifies the relative error between repetitions of the same measurement (Luque Laguna et al., 2020). Thus, high repeatability means that the difference between the repetitions is small compared with the measured value. The ICC quantifies the fraction of variance that is not explained by errors, that is, due to differences between the participants (Matheson, 2019). High reliability means that biological differences between participants can be detected reliably. Like CV_ws_, ICC depends on errors but, unlike CV_ws_, also on differences between participants. Thus, our results for reliability are representative of our sample of participants (10 healthy participants with a mean age of 48 years, standard deviation 14.4 years; 4 women). The results could be different for a different age or sex distribution or in the presence of disease or pathology. It should also be noted that the reproducibility results are affected by the method used to estimate the measures from MD-MRI data (i.e., both the signal processing pipeline and the template-based ROI segmentation).

We investigated the reliability and repeatability of both voxel-based and ROI-averaged MD-MRI parameter estimates. The MD-MRI distribution mean parameters all had median voxel-wise CV_ws_values smaller than 5%, exhibiting good repeatability. Voxel-wise$\text{E}\;[D_{\text{iso}}]$and$\text{E}\;[D_{\Delta }^{2}]$, demonstrated good reliability (ICC = 0.75) and$\text{E}[R_{2}]$moderate reliability (ICC = 0.72). However,$\text{E}[R_{1}]$had poor reliability (ICC<0.50), which may be due to the relatively narrow range of$R_{1}$encoding sensitivity in the acquisition protocol, leading to limited sensitivity. The ROI-averaged distribution means$\text{E}\;[D_{\text{iso}}]$,$\text{E}\;[D_{\Delta }^{2}]$, and$\text{E}[R_{2}]$had high repeatability (CV_ws_< 2%) and reliability (ICC > 0.85).

The diffusion gradient frequency dependency of mean isotropic diffusivity,$\Delta_{\omega /2\pi }\text{ E}[D_{\text{iso}}]$, exhibited poor voxel-wise and ROI-averaged reliability and repeatability.$\Delta_{\omega /2\pi }\text{ E}\;\;[D_{\Delta }^{2}]$had poor reliability but reasonably good repeatability (voxel-wise CV_ws_= 18%, ROI CV_ws_= 7.5%). We believe that these results have two main reasons. First, diffusion gradient hardware limitations constrain the diffusion frequency range to centroid frequencies of 6.6–21 Hz, with the highest frequency achieved only at a low b-value of 0.5 ms/μm^2^, and thus restrict the ability to reliably decompose and estimate the expected time scales in the system. This frequency content range, assuming spherical compartment, zero permeability, and intracellular diffusivity 3.5 μm^2^/ms, is expected to be particularly sensitive to the length scale of approximately 13 µm (Stepišnik, 1993). While this length scale may be characteristic in some brain regions, typical length scales in most of the human brain are significantly smaller. This mismatch between the sensitivity range of the MD-MRI acquisition and the underlying microstructure is a potential driver of the poor reproducibility we found. We believe that the second contributor to these findings is the way in which the frequency-dependence parameters are defined, that is, as a subtraction of two parameters. Thus, they suffer from error propagation as inaccuracies are summated during subtraction. Furthermore, the absolute differences between the highest and lowest frequencies are very small due to the limited frequency range, leading to small numbers around 0, with reduced sensitivity. Based on these results, finding an alternative way to quantify frequency dependence from theD(ω)-$R_{1}$-$R_{2}$distributions is encouraged.

One of the unique features of the MD-MRI framework is the ability to access and visualize intravoxel information within the human brain by selectively integrating regions of the voxel-wiseD(ω)-$R_{1}$-$R_{2}$distributions. Consistent with previous studies, we delineated three*spectral*ROIs in the 2D$D_{\text{iso}}$-$D_{\Delta }^{2}$distribution space, roughly corresponding to WM, GM, and CSF, denoted as*bin1*,*bin2*, and*bin3*, respectively, as illustrated inFigure 2. Voxel-wise application of partial integration based on these bins yields corresponding signal fraction maps, labeled as$f_{\text{bin}1}$,$f_{\text{bin}2}$, and$f_{\text{bin}3}$. Voxel-wise reliability and repeatability analyses resulted in whole brain median ICC values of 0.67, 0.68, and 0.59, and CV_ws_values of 6%, 15%, and 90%, for$f_{\text{bin}1}$,$f_{\text{bin}2}$, and$f_{\text{bin}3}$, respectively. The ROI-based performance was better, with median ICC values ranging from 0.80 to 0.94, and CV_ws_values in the range of 2–11%. We hypothesize that the relatively poor reproducibility of$f_{\text{bin}3}$was due to the small amounts of free water in non-CSF regions in the brain, leading to high relative errors in the estimated signal fractions. This effect is particularly exacerbated when reporting median values across the whole brain, with only a small portion of the voxels residing in CSF-containing regions, as can be seen in the almost binary-looking$f_{\text{bin}3}$map inFigure 4. The overall good reproducibility of the intravoxel fraction maps is encouraging, especially because such subvoxel component maps have demonstrated specificity toward pathology and have been used to characterize subtle processes noninvasively (Benjamini, Iacono, et al., 2021;Benjamini et al., 2022;Slator et al., 2019).

The subvoxel spectral information can be further utilized to resolve relaxation parameters based on their diffusion characteristics, such as the bin-resolved mean relaxation rates$\text{E}{\left[R_{1}\right]}_{\text{bin}1}$,$\text{E}{\left[R_{1}\right]}_{\text{bin}2}$,$\text{E}{\left[R_{1}\right]}_{\text{bin}3}$,$\text{E}{\left[R_{2}\right]}_{\text{bin}1}$,$\text{E}{\left[R_{2}\right]}_{\text{bin}2}$, and$\text{E}{\left[R_{2}\right]}_{\text{bin}3}$, thus directly visualizing diffusion–relaxation correlation. Of those bin-resolved parameters,$\text{E}{\left[R_{1}\right]}_{\text{bin}2}$,$\text{E}{\left[R_{1}\right]}_{\text{bin}3}$,$\text{E}{\left[R_{2}\right]}_{\text{bin}1}$, and$\text{E}{\left[R_{2}\right]}_{\text{bin}2}$had moderate median voxel-wise reliabilities (ICC > 0.5) and good ROI-averaged reliabilities (ICC > 0.75). Their repeatability values were relatively good (voxel-wise CV_ws_4-12%, ROI CV_ws_2-5%), except those of$\text{E}{\left[R_{1}\right]}_{\text{bin}3}$, which were poorer (voxel-wise CV_ws_= 100%, ROI CV_ws_= 16%).$\text{E}{\left[R_{1}\right]}_{\text{bin}1}$had low voxel-wise reliability (ICC = 0.39), moderate ROI reliability (ICC = 0.54), and good repeatability (voxel-wise CV_ws_= 5.5%, ROI CV_ws_= 3.1%). Interestingly,$\text{E}{\left[R_{2}\right]}_{\text{bin}3}$had poor voxel-wise reliability (ICC = 0.29) and repeatability (CV_ws_= 130%) but good ROI-averaged reliability (ICC = 0.81). Taken together, these results suggest that MD-MRI scalar parameters that combine information from different contrasts (diffusion and relaxation) can be reliable and repeatable. However, parameters related to bin3 (CSF) provide a special case to consider. Such parameters had considerably lower, even very poor, median voxel-wise reliability and repeatability values. Averaging those parameters over an ROI greatly increased their reliability and repeatability values. The voxel-wise estimates could have been greatly influenced by the low amount of CSF in most tissues and by inaccuracies in image registration.

Comparing our reliability and repeatability results with previous studies would provide important context. While the current study is the first to report on reproducibility of MD-MRI, we can draw parallels with previous diffusion MRI variability investigations. Specifically, MD and FA in our study align with$\text{E}\;\;[D_{\text{iso}}]$and$\text{E}\;\;[D_{\Delta }^{2}]$, respectively. Most of the recent DTI studies investigated ICC and CV_ws_in WM regions. Repeatability and reliability of DTI metrics in the CC reported, for MD and FA, respectively, CV_ws_ranges of 1–2.5% and 2.5–3%, and ICC ranges of 0.54–0.73 and 0.76–0.98 (Coelho et al., 2022;Fan et al., 2021;Grech-Sollars et al., 2015;Luque Laguna et al., 2020). Our investigation in the CC revealed, for$\text{E}\;[D_{\text{iso}}]$and$\text{E}\;[D_{\Delta }^{2}],$both an average CV_ws_of 1%, and average ICC of 0.88 and 0.91, respectively. In addition to$\text{E}[D_{\text{iso}}]$and$\text{E}\;[D_{\Delta }^{2}]$, the reproducibility of$\text{E}[R_{1}]$and$\text{E}[R_{2}]$can also be compared with relaxometry studies in the CC, in which the CV_ws_was established at under 1% for both$T_{1}$and$T_{2}$(Hagiwara et al., 2019), comparable with our reported values around 2%. Another rough comparison could be drawn between the free water fraction from the so-called Standard Model (Novikov et al., 2019) and MD-MRI derived$f_{\text{bin}3}$. A recent reproducibility study found CV_ws_values of 9% and 15% in the CC and internal capsule, respectively (Coelho et al., 2022), comparable with CV_ws_average values of 10% and 16% we reported here for$f_{\text{bin}3}$. Notably, the reproducibility of MD-MRI diffusion measures in the subcortical regions we investigated surpassed those of reported DTI parameters. For instance, reliability for MD and FA in the basal ganglia (CV_ws_of 4% and 10%) and in the thalamus (CV_ws_of 10% and 8%) (Grech-Sollars et al., 2015) should be compared with the corresponding values of 2% for$\text{E}\;[D_{\text{iso}}]$and 4% for$\text{E}\;[D_{\Delta }^{2}]$reported in our study.

Our study has several limitations and assumptions. The MD-MRI signal representation used provides nonparametric distributions of diffusion and relaxation components and is based on the Gaussian phase distribution (GPD) approximation (Neuman, 1974;Stepišnik, 1981). Non-Gaussian diffusion effects that violate the GPD assumption (Jespersen et al., 2019) and induce microscopic kurtosis are possible (Novello et al., 2022). In this case, the microscopic kurtosis is not accounted for in our model, and its effect would split between variances of$D_{\text{iso}}$and$D_{\Delta }^{2}$. Considering relaxation, quantitative comparisons of$R_{1}$and$R_{2}$across studies are hindered by fundamental considerations in pulse sequence and acquisition design. The recorded signal results from intricate interactions among partial excitation, relaxation processes, and proton pool exchange, each possessing distinct MR properties (Manning et al., 2021). These factors make$R_{1}$and, to a lesser extent,$R_{2}$, dependent on specific pulse sequence parameters, slice thickness, and radiofrequency bandwidth. Furthermore, hardware limitations restricted the minimal echo time to 40 ms in this study, which may fully attenuate myelin water ($R_{2}\sim$100 s^-1^) (Manning et al., 2021). Future improvements in diffusion gradient frequency range, b-values, and echo time minimization could be achieved by utilizing next-generation diffusion gradients (Dai et al., 2023;Huang et al., 2021;Michael et al., 2022). When assessing the reproducibility results, we should keep in mind that the study included 10 participants, and the results related to reliability are representative of that sample. Although consistent with the median sample size of six reported in technical MRI studies (Hanspach et al., 2021), a larger sample would likely yield more robust MD-MRI reproducibility values. Additionally, our focus on intrascanner reproducibility highlights the need for future investigations into interscanner assessment.

While informative, voxel-wise reproducibility analysis requires image registration to a common space, introducing inaccuracies due to biological variation and image interpolation. Although we applied Gaussian spatial filtering to mitigate these effects, complete elimination cannot be achieved. The level of smoothing we applied for our voxel-wise results is consistent with levels of smoothing typically applied (FWHM: 2–4 pixels; 4–10 mm), indicating that our results are representative of this field of research (Caceres et al., 2009;Shou et al., 2013;Somandepalli et al., 2015). However, smoothing is not always applied in voxel-wise analyses (Coelho et al., 2022;Luque Laguna et al., 2020;Veraart et al., 2021). Similar to the voxel-wise analysis, the ROI-based analysis is also affected by the accuracy of the segmentation results (Supplementary Fig. 3). For ROI-based analysis, we first estimated voxel-wise MD-MRI parameters and then averaged them over the ROI, as this approach reduces tissue heterogeneity in the estimation step. Conversely, averaging signals before parameter estimation would increase the expected number of distinct components, potentially affecting accuracy, and was, therefore, avoided.

## 5 Conclusion

We established reliable quantification of relaxation and diffusion properties, including intravoxel information, using a clinically feasible, 40-min*in vivo*diffusion–relaxation correlation (D(ω)-$R_{1}$-$R_{2}$) multidimensional MRI scan in the human brain. This enabled us to methodologically explore brain regions in terms of their diffusion–relaxation multicomponent properties and interpret differences and similarities between them. We were able to observe subtle variations between subcortical and cortical regions, and diversity of WM tracts, demonstrating the microstructural sensitivity of the MD-MRI framework. We further established repeatability and reliability of the pipeline, and showed it to be comparable with known diffusion MRI reproducibility. The possibilities of using the rich spectral information in the MD-MRI data to create biomarkers that show high sensitivity to specific cell types or pathologies should be further investigated. To ensure the reproducibility and adoption of such biomarkers, future studies should also investigate the interscanner reproducibility and the harmonization of MD-MRI datasets.

## Supplementary Material

Supplementary Material

## Data Availability

The data that support the findings of this study are available on request from the corresponding author. The data are not publicly available due to privacy or ethical restrictions. Code to process the MD-MRI data is freely available as implemented in the multidimensional diffusion MRI toolbox (https://github.com/markus-nilsson/md-dmri).
