# Supplementary material for: Variability of multidimensional diffusion–relaxation MRI estimates in the human brain
Source: Imaging Neurosci (Camb). Author manuscript; Available in PMC 2025 Aug 12. (PMC12315732; doi:10.1162/imag_a_00387)
Supplement: SI [file NIHMS2048968-supplement-SI.pdf]

Supplementary information: Variability of multidimensional diffusion-relaxation MRI estimates in the human brain

Eppu Manninen[a], Shunxing Bao[b], Bennett A. Landman[b], Yihong Yang[c], Daniel Topgaard[d], Dan Benjamini[a]

[a]Multiscale Imaging and Integrative Biophysics Unit, National Institute on Aging, NIH, Baltimore, MD, USA
[b]Department of Electrical and Computer Engineering, Vanderbilt University, Nashville, TN, USA
[c]Neuroimaging Research Branch, National Institute on Drug Abuse, Baltimore, MD, USA
[d]Department of Chemistry, Lund University, Lund, Sweden

Corresponding Author: Dan Benjamini (dan.benjamini@nih.gov)

Supplementary Figures

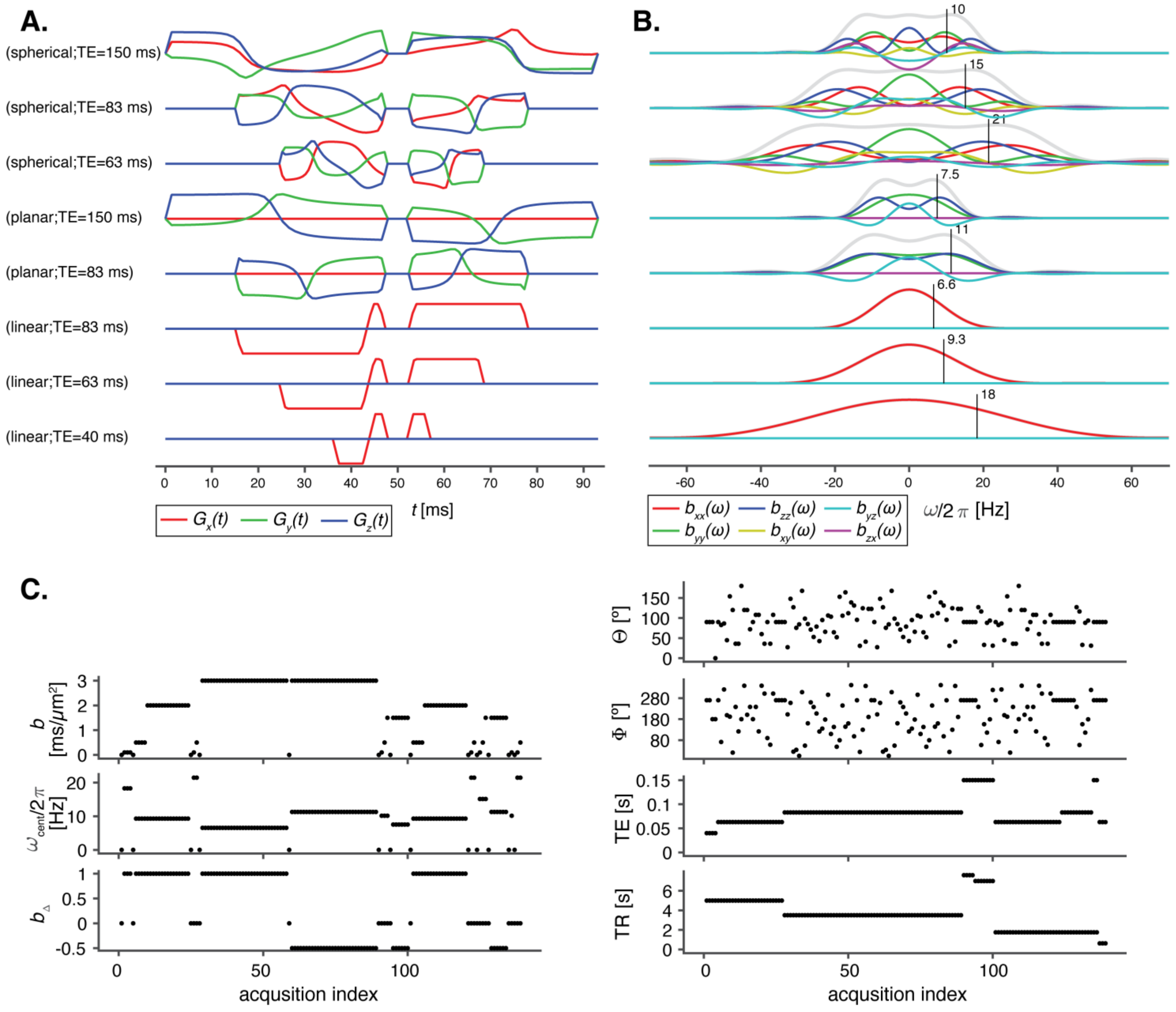

Supplementary Fig. 1. Key experimental details. (A) Time-dependent effective gradients $\mathbf{G}(t)$ and (B) corresponding tensor-valued encoding spectra $\mathbf{b}(\omega)$ for linear, planar, and spherical encoding at different echo times and centroid frequencies, denoted by black vertical lines. (C) Acquisition protocol with repetition time TR, echo time TE, as well as $\mathbf{b}$-tensor magnitude b, normalized anisotropy b_Δ (planar: –0.5, spherical: 0, linear: 1), orientation (θ, ϕ), and centroid frequency $\omega_{\mathrm{cent}}/2\pi$, versus image acquisition index.

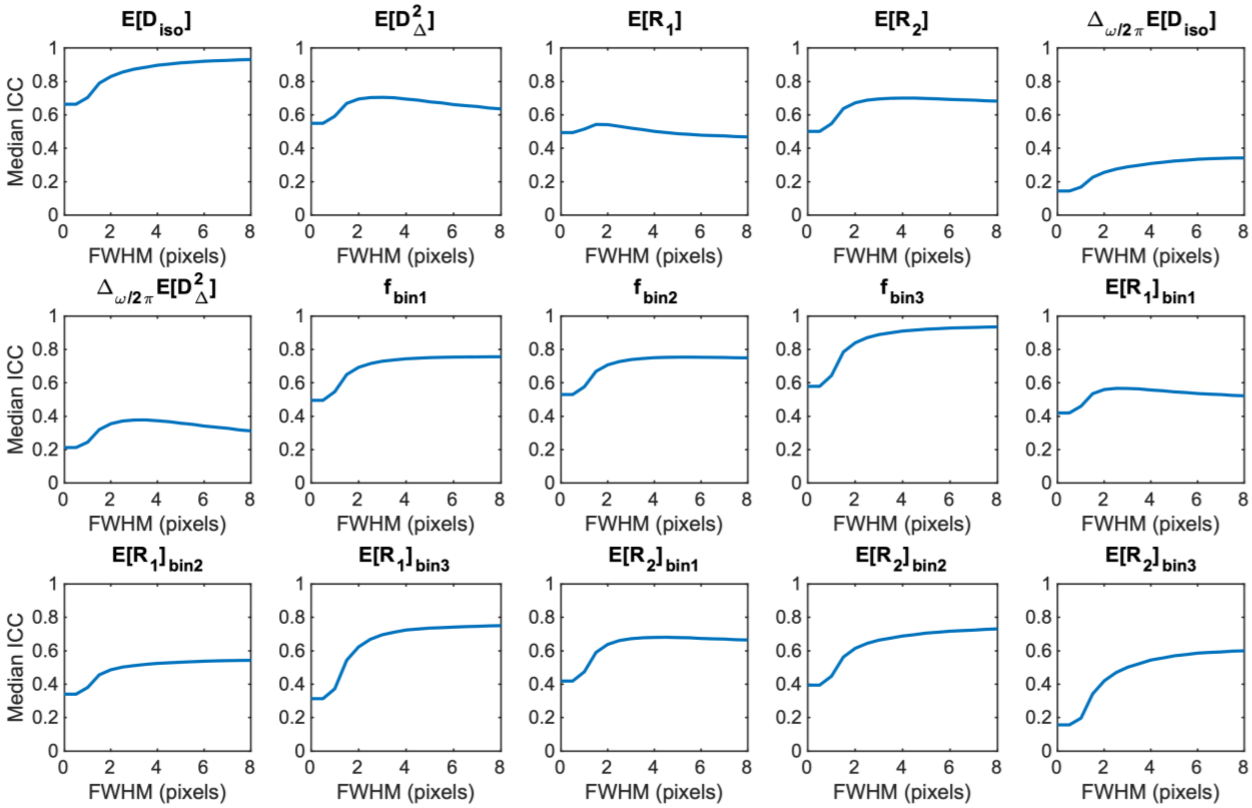

Supplementary Fig. 2. The dependence of median voxel-wise intraclass correlation coefficient (ICC; reliability) on spatial smoothing. The median ICC was computed over all brain voxels in the cohort template space at different values of the full width at half maximum (FWHM) of the Gaussian spatial smoothing kernel. One pixel corresponds to 2 mm.

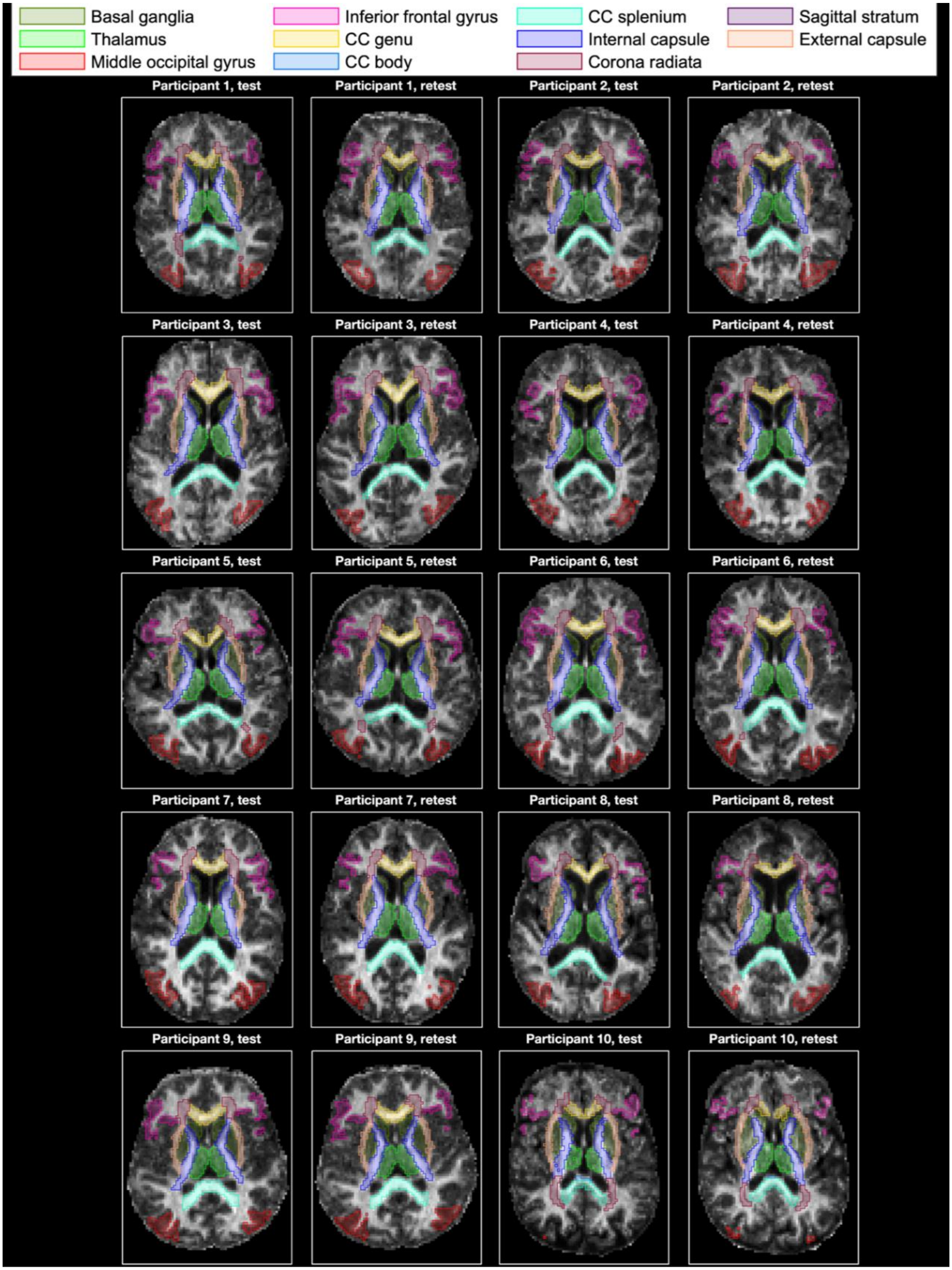

Supplementary Fig. 3. Regions of interest (ROI) in the native space shown for each repetition and participant. The ROIs are overlaid on top of the $\mathrm{E}[D_\Delta^2]$ map.

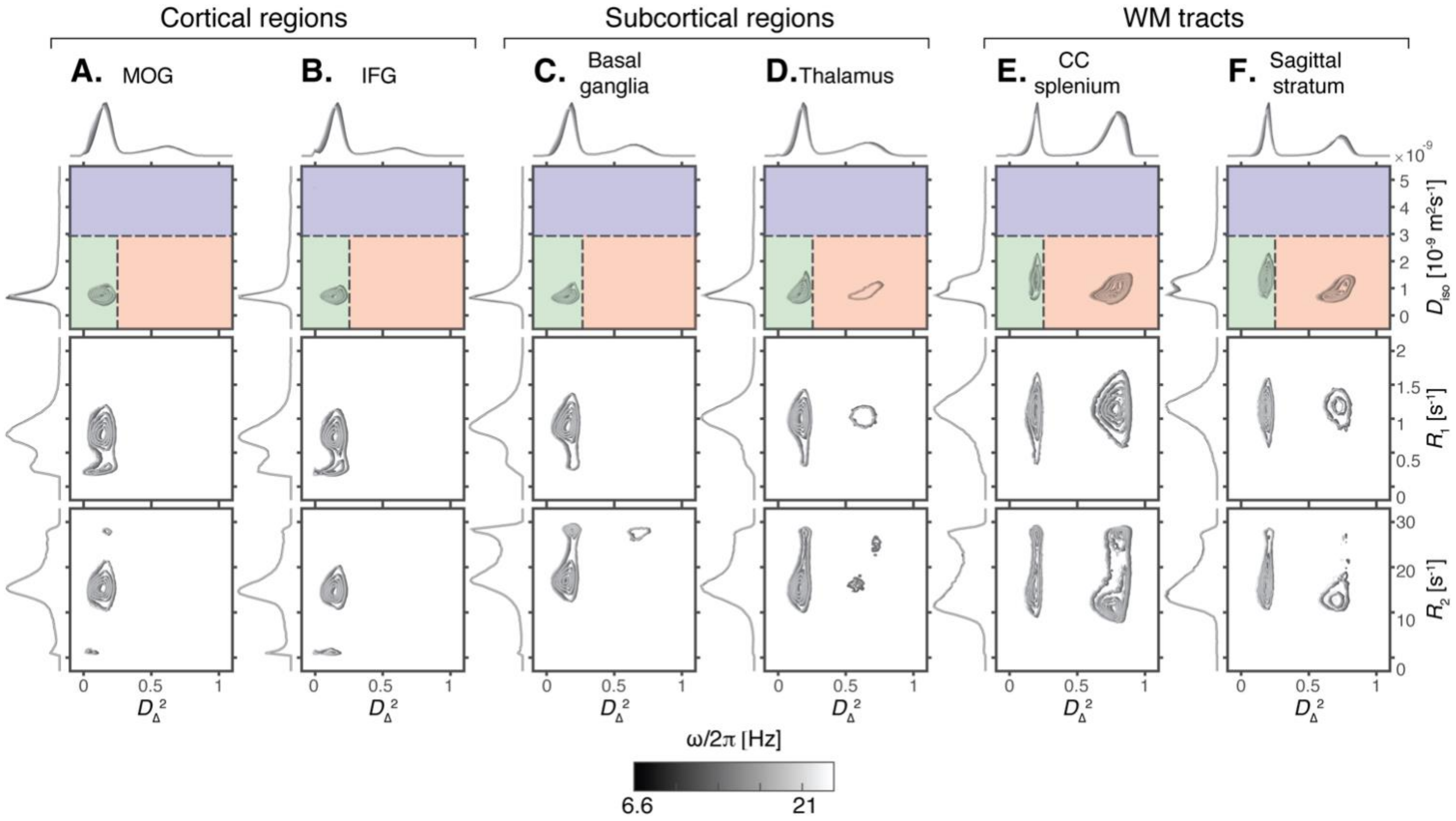

Supplementary Fig. 4. Projections of estimated $\mathbf{D}(\omega)$-R_1-R_2 distributions in different regions of interest (ROI). The displayed 2D projections include D_Δ^2 -D_{iso} (top row), D_Δ^2-R_1 (middle row), and D_Δ^2-R_2 (bottom row) distributions. Different grayscale contour lines represent different centroid frequencies for the diffusion encoding gradients ($\omega/2\pi = 6.6 - 21$ Hz). Colored areas in the D_Δ^2 -D_{iso} distribution plots represent bins that approximately correspond to white matter (red bin), gray matter (green bin), and cerebrospinal fluid (blue bin). Each distribution was averaged over the 10 participants, 2 repetitions, and the different voxels included in each ROI. (A) Middle occipital gyrus (MOG), (B) Inferior occipital gyrus (IFG), (C) Basal ganglia, (D) Thalamus, (E) Splenium of the corpus callosum (CC), (F) Sagittal stratum.

A

Mean

	Basal ganglia	Thalamus	Middle occipital gyrus	Inferior frontal gyrus	CC genu	CC body	CC splenium	Internal capsule	Corona radiata	Sagittal stratum	External capsule
$E[D_{iso}]$ ($\mu m^2/ms$)	0.93	1.01	0.95	1.05	1.11	1.10	1.09	1.03	1.06	1.15	1.01
$E[D_\Delta^2]$	0.32	0.38	0.27	0.25	0.57	0.59	0.67	0.62	0.59	0.52	0.46
$E[R_1]$ (s^{-1})	0.92	0.99	0.76	0.71	1.07	1.05	1.13	1.08	1.06	1.15	0.97
$E[R_2]$ (s^{-1})	20.70	17.90	16.60	15.20	17.00	15.60	16.70	17.50	15.50	16.60	16.70
$\Delta_{\omega/2\pi}E[D_{iso}]$ (μm^2)	1.30	1.00	6.90	0.80	-0.40	0.90	1.20	1.10	1.50	-0.20	-0.10
$\Delta_{\omega/2\pi}E[D_\Delta^2]$ (ms)	-2.01	-2.50	-2.20	-2.00	-2.80	-3.20	-2.40	-2.80	-3.80	-2.70	-2.40
f_{bin1}	0.41	0.51	0.36	0.31	0.72	0.74	0.84	0.80	0.76	0.65	0.60
f_{bin2}	0.56	0.46	0.60	0.62	0.23	0.21	0.12	0.17	0.21	0.30	0.37
f_{bin3}	0.01	0.01	0.03	0.05	0.02	0.02	0.01	0.00	0.00	0.01	0.01
$E[R_1]_{bin1}$ (s^{-1})	0.82	0.96	0.63	0.58	1.09	1.06	1.15	1.09	1.07	1.16	0.98
$E[R_1]_{bin2}$ (s^{-1})	0.88	0.94	0.78	0.74	0.66	0.62	0.41	0.51	0.70	1.03	0.84
$E[R_1]_{bin3}$ (s^{-1})	0.02	0.03	0.06	0.11	0.05	0.05	0.04	0.01	0.01	0.05	0.02
$E[R_2]_{bin1}$ (s^{-1})	20.60	17.90	16.30	15.00	16.70	14.60	16.10	17.00	15.10	15.50	16.90
$E[R_2]_{bin2}$ (s^{-1})	18.40	15.00	15.10	14.30	10.40	9.60	5.70	8.50	9.60	14.10	13.10
$E[R_2]_{bin3}$ (s^{-1})	0.50	0.70	0.80	1.40	1.00	1.00	0.80	0.30	0.10	1.00	0.40

B

SD

	Basal ganglia	Thalamus	Middle occipital gyrus	Inferior frontal gyrus	CC genu	CC body	CC splenium	Internal capsule	Corona radiata	Sagittal stratum	External capsule
$E[D_{iso}]$ ($\mu m^2/ms$)	0.19	0.17	0.20	0.22	0.16	0.17	0.17	0.14	0.10	0.14	0.15
$E[D_\Delta^2]$	0.12	0.12	0.11	0.08	0.13	0.14	0.15	0.15	0.10	0.08	0.12
$E[R_1]$ (s^{-1})	0.12	0.12	0.13	0.12	0.15	0.15	0.14	0.13	0.10	0.13	0.13
$E[R_2]$ (s^{-1})	3.70	2.30	2.30	2.10	2.30	2.20	2.30	2.70	1.90	1.80	2.10
$\Delta_{\omega/2\pi}E[D_{iso}]$ (μm^2)	4.70	5.30	5.90	4.80	4.30	4.20	3.30	3.50	3.80	4.40	4.40
$\Delta_{\omega/2\pi}E[D_\Delta^2]$ (ms)	0.99	1.20	1.20	1.10	1.50	1.90	1.60	1.50	1.80	1.30	1.20
f_{bin1}	0.19	0.18	0.18	0.15	0.19	0.20	0.18	0.20	0.16	0.13	0.18
f_{bin2}	0.20	0.18	0.16	0.14	0.18	0.19	0.16	0.19	0.15	0.13	0.18
f_{bin3}	0.04	0.04	0.06	0.07	0.04	0.04	0.04	0.02	0.01	0.03	0.03
$E[R_1]_{bin1}$ (s^{-1})	0.22	0.17	0.25	0.23	0.17	0.16	0.14	0.14	0.11	0.14	0.17
$E[R_1]_{bin2}$ (s^{-1})	0.18	0.21	0.13	0.12	0.44	0.47	0.49	0.47	0.43	0.29	0.25
$E[R_1]_{bin3}$ (s^{-1})	0.07	0.12	0.13	0.16	0.15	0.17	0.15	0.06	0.06	0.17	0.08
$E[R_2]_{bin1}$ (s^{-1})	4.60	2.60	4.70	4.40	2.30	2.30	2.40	2.90	2.10	2.00	2.30
$E[R_2]_{bin2}$ (s^{-1})	4.50	4.00	2.00	1.90	6.60	7.10	6.90	7.80	5.90	4.10	4.00
$E[R_2]_{bin3}$ (s^{-1})	2.60	2.90	2.60	3.00	3.40	3.30	2.90	1.80	1.30	3.50	2.00

Supplementary Fig. 5. (A) Mean and (B) standard deviation (SD) of each MD-MRI parameter map, computed over all participants, repetitions, and voxels for each region of interest (ROI). Color scaling is normalized separately for each parameter map (row; darker blue is higher) to enable comparison between ROIs for each map. Abbreviations: CC, corpus callosum.

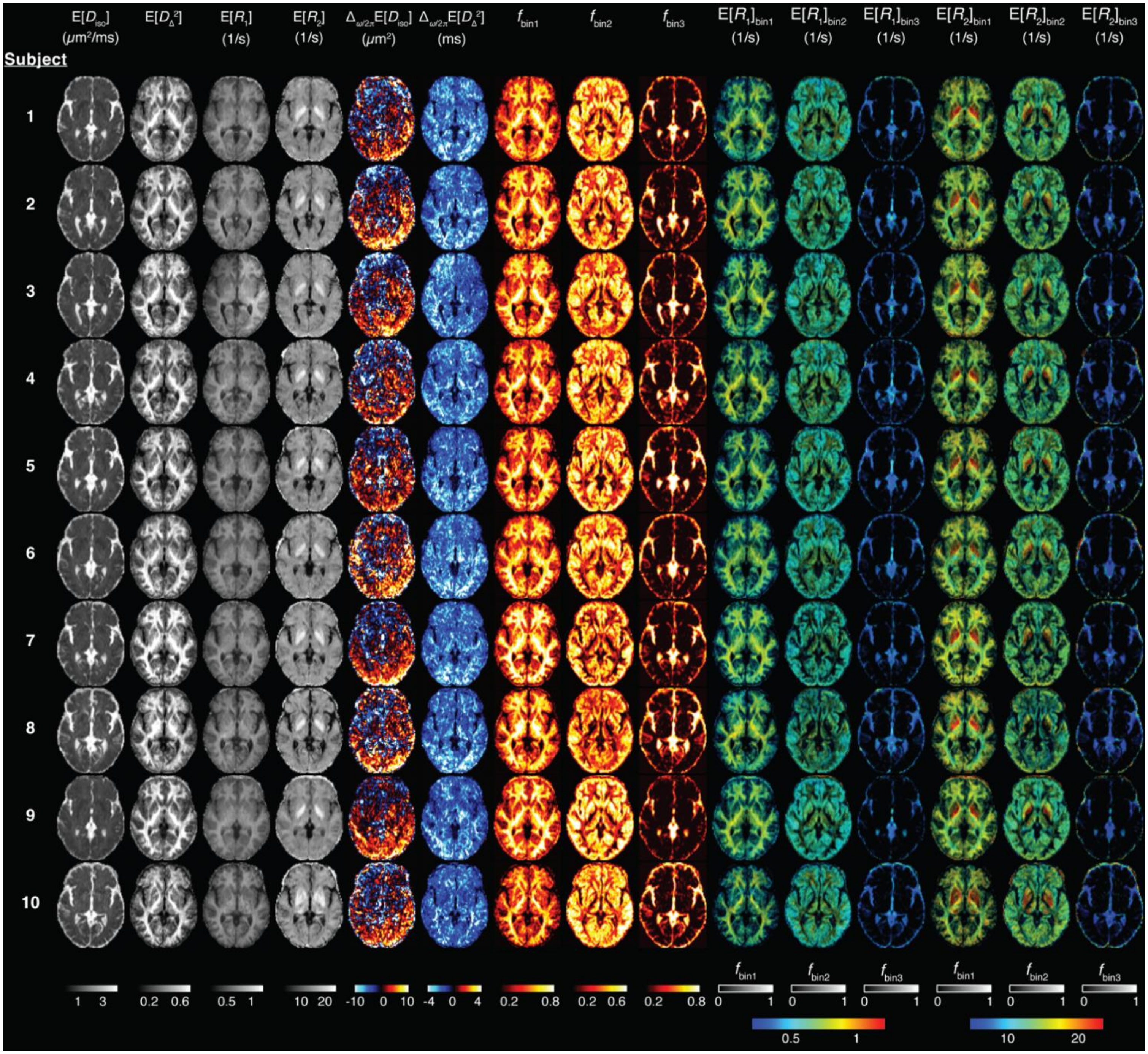

Supplementary Fig. 6. Parameter maps from all participants (first scan repetition).

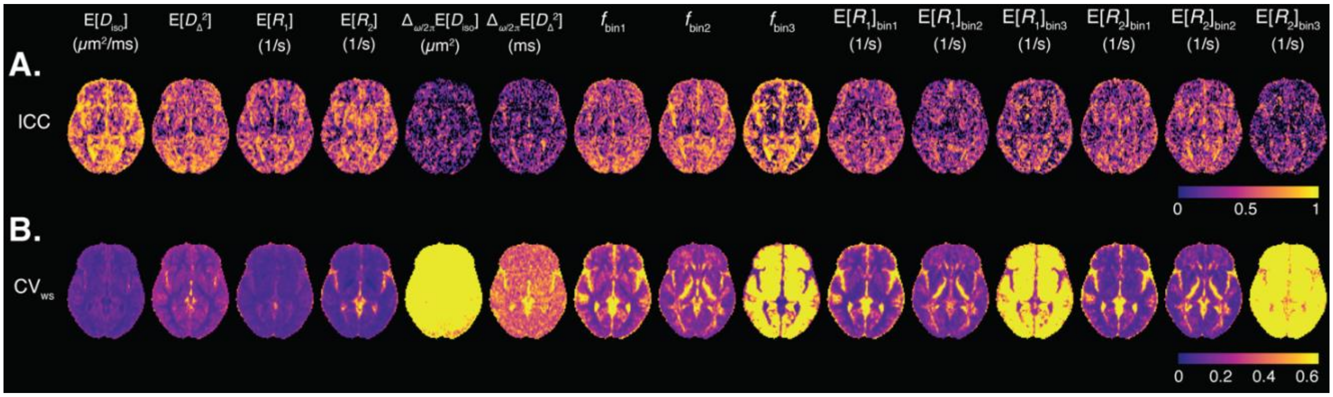

Supplementary Fig. 7. Voxel-wise intraclass correlation coefficient (ICC; reliability) and within-subject coefficient of variation (CV_{ws}; repeatability) without the application of spatial filtering to the maps.

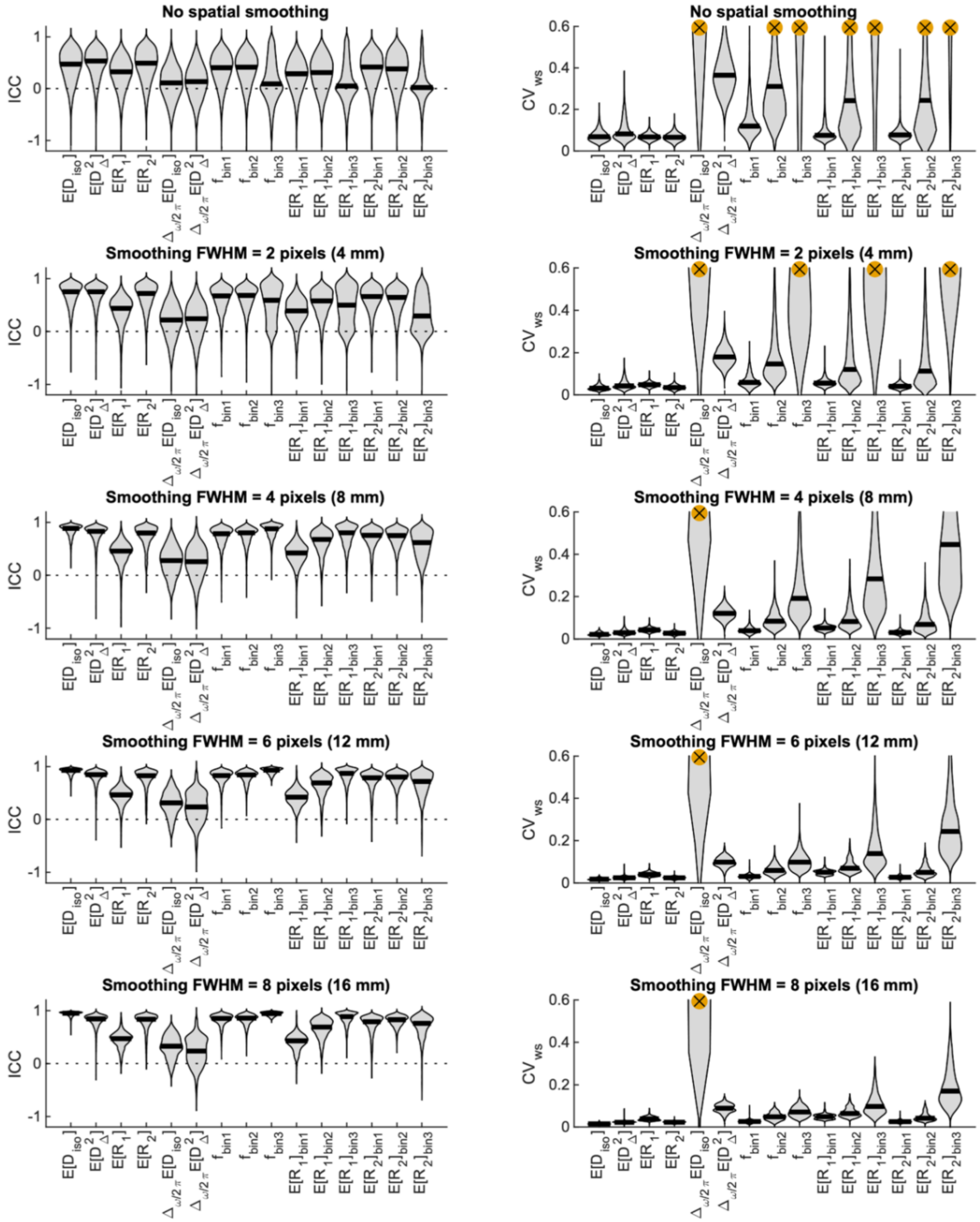

Supplementary Fig. 8. Distributions and medians of voxel-wise reliability and repeatability measures of MD-MRI parameter maps over the whole brain. The results are presented with different levels of spatial filtering (Gaussian kernel) applied to the voxel-wise parameter maps before the computation of the reliability and repeatability measures. (A) Intraclass correlation coefficient (ICC; reliability). (B) Within-subject coefficient of variation (CV_{ws}; repeatability). Some of the distributions included severe outliers that hindered the visualization. Such outliers were removed, and the affected distributions were marked with crosses (see main text).

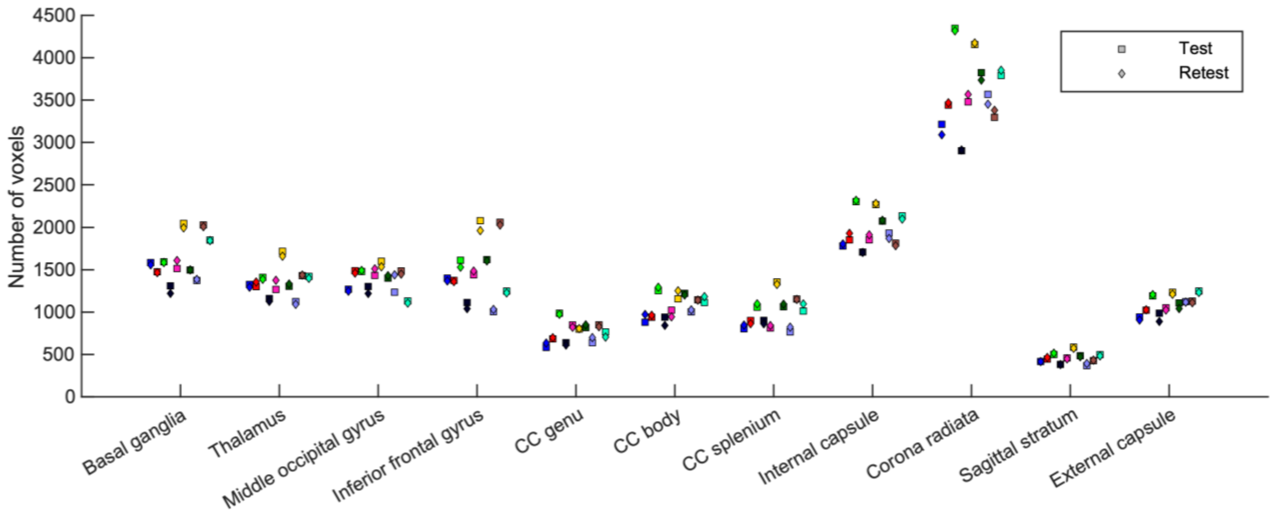

Supplementary Fig. 9. Number of voxels for each region of interest (ROI), participant, and repetition. Different colors represent different participants. Repetition 1 (test) is indicated with a square and repetition 2 (retest) with a diamond marker. Abbreviations: CC, corpus callosum.

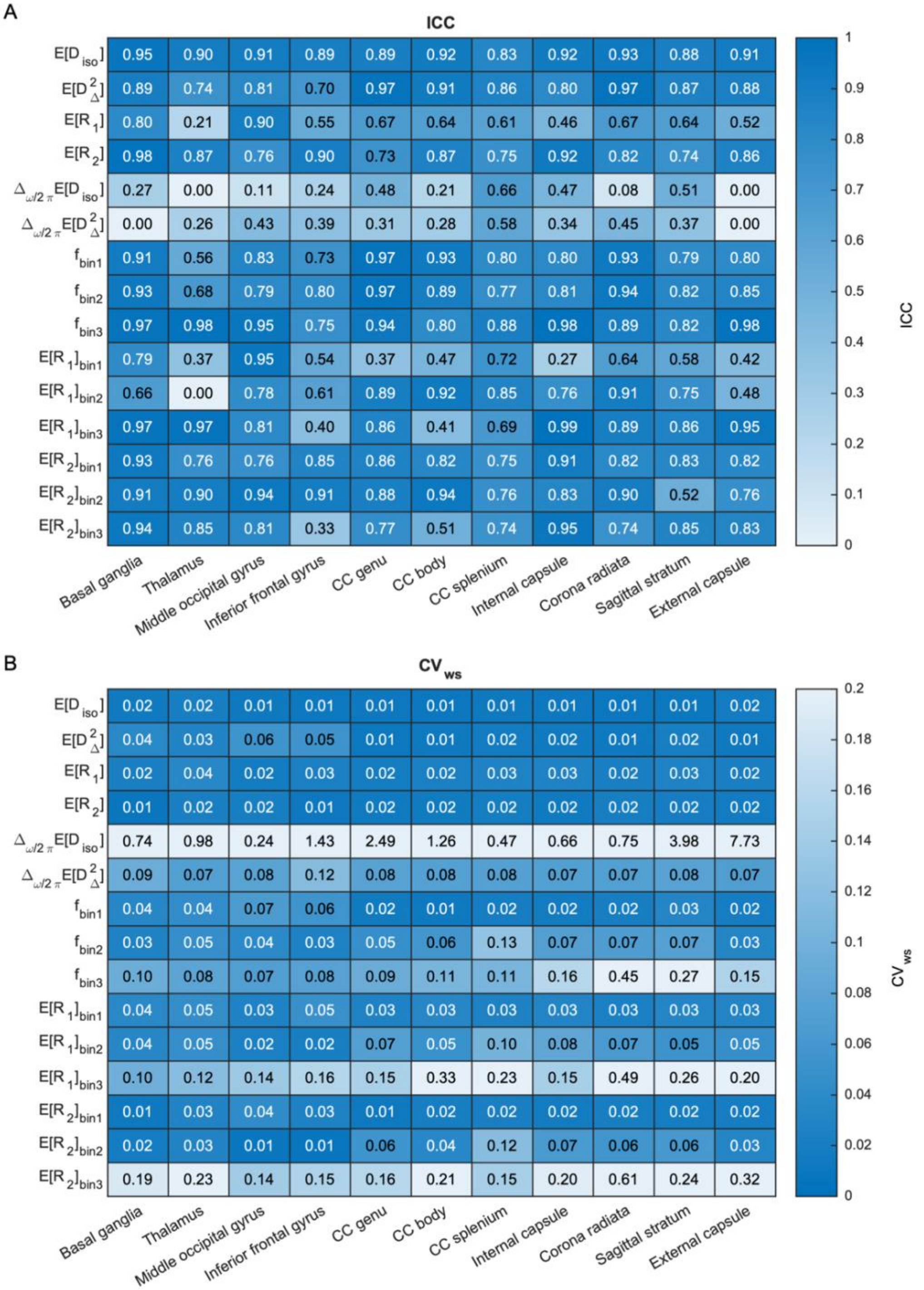

Supplementary Fig. 10. Reliability and repeatability of region-of-interest-averaged MD-MRI parameter maps. (A) Intraclass correlation coefficient (ICC; reliability). (B) Within-subject coefficient of variation (CV_{ws}; repeatability). Generally, ROI-based ICC values were higher and CV_{ws} values lower than those of individual voxels (Figures 1 and 2). That was expected due to the increase in signal-to-noise ratio achieved through averaging. Abbreviations: CC, corpus callosum.
